# Supplementary material for: Sexual health needs of female sex workers in Côte d’Ivoire: a mixed-methods study to prepare the future implementation of pre-exposure prophylaxis (PrEP) for HIV prevention
Source: BMJ Open. 2020 Jan 8;10(1):e028508. doi: 10.1136/bmjopen-2018-028508 (PMC6955511; doi:10.1136/bmjopen-2018-028508)
Supplement: Author's manuscript [file bmjopen-2018-028508.draft_revisions.pdf]

BMJ Open

BMJ Open is committed to open peer review. As part of this commitment we make the peer review history of every article we publish publicly available.

When an article is published we post the peer reviewers' comments and the authors' responses online. We also post the versions of the paper that were used during peer review. These are the versions that the peer review comments apply to.

The versions of the paper that follow are the versions that were submitted during the peer review process. They are not the versions of record or the final published versions. They should not be cited or distributed as the published version of this manuscript.

BMJ Open is an open access journal and the full, final, typeset and author-corrected version of record of the manuscript is available on our site with no access controls, subscription charges or pay-per-view fees (http://bmjopen.bmj.com).

If you have any questions on BMJ Open's open peer review process please email info.bmjopen@bmj.com

BMJ Open

Sexual health needs of female sex workers reached by two NGOs in Côte d'Ivoire: considerations for the future implementation of PrEP

Journal:	*BMJ Open*
Manuscript ID	bmjopen-2018-028508
Article Type:	Original research
Date Submitted by the Author:	11-Dec-2018
Complete List of Authors:	Becquet, Valentine; Centre Population et Développement, Institut de Recherche pour le Développement, Université Paris Descartes, ERL Inserm U 1244<br>Nouaman, Marcellin; Programme PAC-CI/ANRS Research Site<br>Plazy, Mélanie; Inserm Research Center 1219 (Bordeaux Population Health), ISPED, Université de Bordeaux<br>Masumbuko, Jean-Marie; Programme PAC-CI/ANRS Research Site<br>Anoma, Camille; Espace Confiance<br>Kouame, Soh; Aprosam<br>Danel, Christine; Programme PAC-CI/ANRS Research Site<br>Eholie, Serge; Programme PAC-CI/ANRS Research Site<br>Larmarange, Joseph; Centre Population et Développement, Institut de Recherche pour le Développement, Université Paris Descartes, ERL Inserm U 1244
Keywords:	QUALITATIVE RESEARCH, HIV & AIDS < INFECTIOUS DISEASES, Public health < INFECTIOUS DISEASES, Demography < TROPICAL MEDICINE, Epidemiology < TROPICAL MEDICINE

SCHOLARONE™
Manuscripts

1. Title page

Sexual health needs of female sex workers reached by two NGOs in Côte d'Ivoire: considerations for the future implementation of PrEP

Valentine Becquet[1§], Marcellin Nouaman[2], Mélanie Plazy[3], Jean-Marie Masumbuko[2], Camille Anoma[4], Soh Kouamé[5], Christine Danel[2], Serge Eholié[2], Joseph Larmarange[1] for the ANRS 12361 PrEP-CI Study group

[1]Centre Population et Développement, Institut de Recherche pour le Développement, Université Paris Descartes, ERL Inserm U 1244, Paris, France

[2]Programme PAC-CI site ANRS de Côte d'Ivoire, Abidjan, Côte d'Ivoire

[3]Inserm Research Center 1219 (Bordeaux Population Health), ISPED, Université de Bordeaux, Bordeaux, France

[4]Espace Confiance, Abidjan, Côte d'Ivoire

[5]Aprosam, San Pedro, Côte d'Ivoire

§ Correspondance to Valentine Becquet, PhD, Centre Population et Développement, Université Paris Descartes, 45 rue des Saints-Pères, 75006 Paris, France. +33660082287

Email addresses:
VB: valentine.becquet@gmail.com
MN: nouaman_et_vie@yahoo.fr
MP: melanie.plazy@u-bordeaux.fr
JMM: masumbukojm@gmail.com
CA: anomacamille@yahoo.fr
SK: sohkouame1@yahoo.fr
CD: christinemarie.danel@gmail.com
SE: sergeholie@yahoo.fr
JL: joseph.larmarange@ceped.org

Members of the PrEP-CI study group:

- ABOUBAKAR Sangaré (Aprosam, San Pedro, Côte d'Ivoire)
- ANGLARET Xavier (PAC-CI, Abidjan, Côte d'Ivoire / Inserm, Bordeaux, France)
- ANOMA Camille (Espace Confiance, Abidjan, Côte d'Ivoire)
- BARIN Francis (Université François Rabelais, Tours, France)
- BAZIN Brigitte (ANRS, Paris, France)
- BECQUET Valentine (Ceped/IRD, Paris, France)
- DABIS François (ISPED/Inserm, Bordeaux, France)
- DANEL Christine (PAC-CI, Abidjan, Côte d'Ivoire / Inserm, Bordeaux, France)
- EHOLIE Serge (PAC-CI, Abidjan, Côte d'Ivoire)
- EKOUEVI Didier (PAC-CI, Abidjan, Côte d'Ivoire)
- FONSART Julien (Hôpital Saint-Louis, Paris, France)
- GBOSI Kate (Aprosam, San Pedro, Côte d'Ivoire)
- KWAMÉ Abo (Programme National de Lutte contre le Sida, Côte d'Ivoire)
- LARMARANGE Joseph (Ceped/IRD, Paris, France)
- MASUMBUKO Jean-Marie (PAC-CI, Abidjan, Côte d'Ivoire)
- MÉDA Nicolas (Centre Muraz, Bobo-Dioulasso, Burkina Faso)

For peer review only - http://bmjopen.bmj.com/site/about/guidelines.xhtml

» MOH Raoul (PAC-CI, Abidjan, Côte d'Ivoire)
» MOLINA Jean-Michel (Hôpital Saint-Louis, Paris, France)
» N'DRI-YOMAN Thérèse (PAC-CI, Abidjan, Côte d'Ivoire)
» NOUAMAN Marcellin (PAC-CI, Abidjan, Côte d'Ivoire)
» PLAZY Mélanie (ISPED / Inserm, Bordeaux, France)
» SOH Kouamé (Aprosam, San Pedro, Côte d'Ivoire)
» TANOE Solange (Espace Confiance, Abidjan, Côte d'Ivoire)
» YEO Roselyne (Espace Confiance, Abidjan, Côte d'Ivoire)

Word count : 4155

For peer review only

For peer review only - http://bmjopen.bmj.com/site/about/guidelines.xhtml

2. Keywords (6)

HIV prevention; sexual and reproductive health; people-focused approach; pre-exposure prophylaxis (PrEP); sex work; mixed-methods research

3. Abstract

Objectives

In West Africa, most countries have mixed HIV epidemics; new tools such as pre-exposure prophylaxis (PrEP) should target in priority most affected populations, particularly female sex workers (FSWs). This paper describes sexual and reproductive health (SRH) needs of FSWs in Côte d'Ivoire to inform the future implementation of PrEP in this population.

Methods

The ANRS 12361 PrEP-CI cross-sectional and mixed-methods study was designed and implemented with two Ivorian community-based organizations.

1000 FSWs completed a standardized questionnaire assessing women's sociodemographic characteristics, sexual practices and behaviors, use of community health services, *a priori* acceptability of PrEP.

Twenty-two in-depth interviews and eight focus group discussions were conducted at prostitution sites. FSWs were interviewed about risky practices and sexual behaviors, experiences with violence and discrimination, attitudes regarding HIV and sexually transmitted infections (STIs), and barriers to SRH services.

Results

FSWs were highly exposed to HIV despite their use of condoms with clients. The large majority did not use condoms with their regular partner, despite their acknowledged concurrent sexual partnerships. Some accepted condomless sexual intercourse for a large sum of money.

Moreover, FSWs faced many unmet needs regarding SRH beyond HIV prevention and treatment. Inconsistent condom use exposed FSWs to STIs and undesired pregnancies. However, the prevalence of contraceptive use was low due to fear of contraception causing sterility. FSWs faced obstacles in accessing SRH care and preferred advice from their peers or self-medication.

Conclusions

Implementing PrEP among FSWs in West Africa, such as in Côte d'Ivoire, constitutes an opportunity to consider the chronic follow-up of HIV-negative FSWs. PrEP initiation should not condition access to SRH services; conversely, SRH services could be a way to attract FSWs into practicing HIV prevention. Our results highlight the importance of developing a people-focused approach that integrates all SRH needs when transitioning from PrEP efficacy trials to implementation.

4. Article Summary

Strengths and limitations of this study

- Mixed-method study that allows to describe and better understand challenges of implementing PrEP among FSWs
- Strong collaboration with NGOs to reach FSWs who participated in the study
- Risk of selection bias as included FSWs, recruited through NGOs, could be more likely to know about HIV prevention and to access SRH care

For peer review only - http://bmjopen.bmj.com/site/about/guidelines.xhtml

5. Main text

INTRODUCTION

Numerous recent efficacy trials have shown highly conclusive results of pre-exposure prophylaxis (PrEP) as an HIV prevention strategy, with a significant reduction in HIV acquisition among men who have sex with men (MSMs), injecting drug users, transsexual women and serodiscordant couples worldwide (1–5). Since 2015, oral PrEP has been recommended by the World Health Organization (WHO) for populations at substantial risk of contracting HIV (6). However, the WHO noted the need for additional operational and social sciences research on creating demand for oral PrEP; improving adherence; understanding the social and behavioral impact of PrEP; and integrating PrEP services with other services (6).

In West Africa, most countries have mixed HIV epidemics, with a relatively low prevalence in the general population (compared to that in Eastern and Southern Africa) but severely affected key populations, particularly female sex workers (FSWs) and MSMs (7). In Côte d'Ivoire, the prevalence of HIV was estimated to be 29% in FSWs in 2012 (8) and 19% among MSMs (8). The National Program against HIV/AIDS (PNLS) requested operational research and data on the relevance of PrEP in order to better consider its future integration in the national algorithm of HIV prevention. A PrEP demonstration project (ANRS 12324 CohMSM) is currently being implemented among MSMs. Our research team was invited to explore the situation among FSWs in this country as a potential target for a PrEP program, knowing that PrEP was not yet available.

Although PrEP is effective when taken properly, the FEM-PREP (9) and VOICE (10) trials conducted among women from the general population in Southern and Eastern Africa showed low adherence to the treatment, resulting in a low or even null effect of PrEP. Similarly, PrEP implementation trials conducted among FSWs in Africa showed varying results regarding retention. In Benin, the retention rate after 10 months was 66% (11) and the overall retention rate after a complete follow-up of 28 months was 48% (12). In South Africa, it was 22% after 12 months (13) despite a high declared acceptability of PrEP before the implementation (14). Moreover, PrEP constitutes a new HIV prevention tool but does not prevent sexually transmitted infections (STIs) or unwanted pregnancies. It is therefore necessary to consider the overall needs of target populations in terms of sexual and reproductive health (SRH) (15).

In this context, in order to design a future PrEP program targeting FSWs, the ANRS 12361 PrEP-CI pilot study was implemented to explore additional needs that should be considered within such a program and to better describe FSWs currently reached by peer educators. This paper aims to describe the work and social environment of FSWs, their SRH needs and possible barriers for accessing care in two different settings in Côte d'Ivoire.

METHODS

Study setting

The ANRS 12361 PrEP-CI cross-sectional and mixed-methods study was designed and implemented with two Ivorian community-based organizations between September 2016 and March 2017. Aprosam works within the city of San Pedro and in the surrounding areas, particularly in villages close to farming businesses (coffee and cocoa exploitation). Espace Confiance operates in several districts of Abidjan, the economic capital of Côte d'Ivoire (Koumassi, Marcory, Treichville, Zone 4 and Port-Bouët with its beaches). Both of these nongovernmental organizations (NGOs) deliver HIV prevention and testing

For peer review only - http://bmjopen.bmj.com/site/about/guidelines.xhtml

services directly at prostitution sites (outreach activities) and provide HIV and SRH care services through a community clinic, for MSMs and FSWs.

Quantitative analysis of a survey questionnaire

From October 2016 to January 2017, a convenient sample of 1000 FSWs completed a 45-item standardized paper questionnaire that assessed their sociodemographic characteristics (age, nationality, level of education, number of children), their sexual practices and behaviors (duration and location of sex work, usual price of sexual intercourses, condom use with clients and regular partners, assault/coerced sexual intercourse), their knowledge and use of community health services (medical consultations, hepatitis B immunization, declared STIs, sex work during menstruations, use and knowledge of contraception, undesired pregnancies, abortion), *a priori* acceptability of a PrEP offer (perception of the risk to contract HIV, knowledge of any medicine (traditional or modern) to prevent HIV infection, interest in a modern medicine for HIV prevention, acceptance of a medical follow-up every three months), HIV infection (HIV testing, knowledge of HIV status of the regular partner).

The purpose of the quantitative study was not to be representative of all FSWs in Côte d'Ivoire but rather to represent FSWs who could be reached by the two partner NGOs and who could potentially benefit from PrEP in a future program. Therefore, the eligibility criteria for the quantitative survey included being 18 years or older, working at a prostitution site at the time of the survey, and never being tested for HIV or previously testing HIV-negative, as we performed a test to determine how many women were exposed to HIV and not diagnosed. In case of a positive result, in order to calculate incidence among this population, dried blood spot (DBS) sampling was performed to determine the window of infection through a recent infection testing algorithm adapted to the Ivorian context. FSWs visiting community clinics of the two NGOs for an HIV test were also included.

Recruitment of participants for this study was made possible by the Aprosam and Espace Confiance organizations' networks of peer educators and their access to the population. Peer educators enrolled FSWs who met eligibility criteria and agreed to participate, after reading an information sheet and signing a consent form. They carried out data collection in dedicated health centers and prostitutions sites during face-to-face interviews.

We compared the sociodemographic characteristics, sexual behaviors and reproductive health of participants surveyed in Abidjan and San Pedro. Due to the fact that it is not exactly a randomly taken sample but rather a convenient sample of women reached by the two NGOs, statistical tests such as Pearson's chi-square test or Fisher's exact test could not be formally used and were therefore not reported. Missing data were excluded from percentage calculations. All analyses were performed with Stata software (StataTM 12.0 College Station, Texas, USA).

Qualitative analysis of interviews

In addition to the quantitative survey, a qualitative study was conducted from November 2016 to December 2016 among a convenient sample of 66 FSWs recruited during the outreach activities of peer educators and with the aim to reach a maximum of different profiles of women (in terms of age, number of years working as FSWs, type of prostitution site).

Data were collected by a sociodemographer who carried out 22 in-depth interviews and eight focus group discussions (FGDs) that took place at prostitution sites in and around Abidjan and San Pedro. FSWs were interviewed about their sociodemographic characteristics (age, nationality, level of education, number of children, number of dependents, partner/husband), sex work (entry into prostitution, duration and location of sex work, usual price of sexual intercourses, mobility, regular clients, work during menstruations, future perspectives), risky practices and sexual behaviors (condom use depending on the type of practices, main perceived risk of unprotected sex, current possession of condoms, negotiation of (un)protected sex with clients and regular partners), community dynamics (relationships with the pimp, the owner of the prostitution site, other FSWs, peer educators from

For peer review only - http://bmjopen.bmj.com/site/about/guidelines.xhtml

NGOs, source of help in case of money, health or administrative issues), experiences with violence and discrimination (physical/moral violence from clients, partners, authorities, experiences of stigmatization, barriers in accessing health care or administrative procedures), knowledge and attitudes regarding HIV and STIs (perception of the risk to contract HIV depending on the type of practices, perception of the global risk to contract HIV and means used for prevention, frequency and location of HIV testing, physical signs of STIs and means used for treatment) , barriers to health care (untreated health issues, locations of care seeking), use of drugs and alcohol, barriers to and need for SRH services (knowledge and screening of cervical cancer, hepatitis B and C, tuberculosis, knowledge and use of contraception including emergency contraception, knowledge and recourse to social workers), *a priori* acceptability of a PrEP offer (knowledge of any medicine (traditional or modern) to prevent HIV infection, interest in a modern medicine for HIV prevention, acceptance of a medical follow-up every three months, issues arisen after the presentation of PrEP) .

Each in-depth interview and FGD was transcribed and uploaded into NVivo software (QSR International Pty Ltd. Version 11 Pro, 2016). The qualitative analysis followed two principles. First, a cross-sectional review, based on questions derived from the discussion guide, allowed for a thematic analysis. Main themes explored for this article were related to the access to care (visit of community health centers, use of mobile clinics, referral by peer educators, barriers in access to care and stigmatization) and to the potential interest, utility of PrEP and obstacles (unprotected sexual intercourses with clients and partners, risk perception and women's priorities, mobility and working periods, access to condoms, use of contraception, anticipation of high-risk sex and violent clients). We remained open to new themes as they emerged from the data in an inductive manner (e.g., condom breakage and self-medication). Second, we reviewed each interview or FGD as a whole in order to identify the chain of events leading each woman to not access healthcare or to not use condoms, for example. Interviews were translated verbatim from French to English by the authors.

Patient and public involvement

No patients were involved in the research design nor in the conduct of the study. Peer educators of six different community NGOs were involved in the development of the research questions during a workshop. The two selected NGOs in Abidjan and San Pedro participated in the design, recruitment and conduct of the study. Data from the quantitative survey and qualitative interviews were disseminated among the community through peer educators, who helped in the interpretation of results.

Ethical considerations

Research authorizations were obtained from the National Committee of Research Ethics within the Ivorian Ministry of Health and Public Hygiene (reference number: 057/MSHP/CNER-kp, delivered on June 28, 2016). Confidentiality was maintained, and data were anonymized. Informed consent was obtained by the investigator before each interview or questionnaire.

RESULTS

Main characteristics of participants

The characteristics of FSWs who participated in the quantitative survey are presented in Table 1. The median age was 25 (IQR=22-30) years in San Pedro and 24 (IQR=21-28) years in Abidjan. Compared to FSWs reached in Abidjan, those reached in San Pedro were less educated, more often Ivorian, more likely to be the mother of at least one child, were paid less money, and they worked less regularly but much more frequently in more than one city. FSWs in San Pedro were also more often in a relationship, and the interviews showed that their boyfriend was often their pimp.

For peer review only - http://bmjopen.bmj.com/site/about/guidelines.xhtml

Peer-educators conducted the survey during their on-site activities, which already consistently extended their working time. For logistic reasons, it was not possible to monitor the number of FSWs present on site, potentially eligible, examined for eligibility and included in the survey. Therefore we are not able to provide participation rates. However, peer educators reported that most FSWs confirmed for eligibility did accept to answer the questionnaire. The survey was stopped when we reached the expected number of 1000 FSWs (600 in Abidjan and 400 in San Pedro) and all FSWs included in the survey were analyzed.

Out of 66 interviewed FSWs, 26 agreed to provide their age: the median age was 28 (IQR=22-33) years. It became evident during the interview process that three FSWs were underage (<18). The interview participants were mostly Ivorian (n=44); the remaining third (n=22) was Nigerian. On each prostitution site visited, we decided to perform in-depth interviews or focus group discussions, depending on the practicality of the site (ambient noise, opportunity of privacy) and the time allowed. Every time, according to the type of interviews to be performed, one to three FSWS agreed to be interviewed individually or five to eight FSWs agreed to be interviewed as a group. Only two participants refused to be recorded as they were afraid to be recognized.

Table 1. Main characteristics of participants in the quantitative survey

Variables	All women n (%) n=1000	San Pedro n (%) n=400	Abidjan n (%) n=600
**Median age (IQR) years**	25 [21 – 29]	25 [22 – 30]	24 [21 – 28]
**Age (years)**			
≤ 24	470 (47.0)	168 (42.0)	302 (50.3)
[25 – 34]	431 (43.1)	181 (45.2)	250 (41.7)
≥ 35	99 (9.9)	51 (12.8)	48 (8.0)
**Level of education**			
No school	220 (22.1)	115 (28.9)	105 (17.6)
Primary school	382 (38.4)	163 (40.9)	219 (36.7)
Secondary school/University	393 (39.5)	120 (30.2)	273 (45.7)
Missing	*5*	*2*	*3*
**Nationality**			
Ivorian	690 (69.0)	312 (78.0)	378 (63.0)
Foreign	310 (31.0)	88 (22.0)	222 (37.0)
**Has a boyfriend/husband**			
Yes	714 (71.9)	317 (80.7)	397 (66.2)
No	279 (28.1)	76 (19.3)	203 (33.8)
Missing	*7*	*7*	*0*
**Number of children**			
0	426 (43.1)	132 (33.3)	294 (49.7)
1	301 (30.5)	122 (30.8)	179 (30.2)
2	155 (15.7)	82 (20.7)	73 (12.3)
≥ 3	106 (10.7)	60 (15.2)	46 (7.8)
Missing	*12*	*4*	*8*
**Frequency of sex work**			
Every day or almost every day	743 (75.3)	275 (69.3)	468 (79.5)
Sometimes	243 (24.7)	122 (30.7)	121 (20.5)
Missing	*14*	*3*	*11*
**How many years sex work has been practiced**			
≤ 2	479 (47.9)	176 (44.0)	303 (50.5)
≥ 3	521 (52.1)	224 (56.0)	297 (49.5)
**Practiced sex work in more than one city**			
Yes	268 (26.9)	198 (49.7)	70 (11.7)
No	727 (73.1)	200 (50.3)	527 (88.3)
Missing	*5*	*2*	*3*
**Where/how clients are contacted[1]**			
Brothel	302 (30.2)	114 (28.5)	188 (31.3)
Beach	129 (12.9)	71 (17.7)	58 (9.7)
Bar/“maquis”	471 (47.1)	200 (50.0)	271 (45.2)

For peer review only - http://bmjopen.bmj.com/site/about/guidelines.xhtml

Street	145 (14.5)	47 (11.8)	98 (16.3)
By phone (through hotel owners)	216 (21.6)	123 (30.7)	93 (15.5)
Hotel	265 (26.5)	156 (39.0)	109 (18.2)
Home	131 (13.1)	66 (16.5)	65 (10.8)
**Number of clients during last day of work**			
≤ 4	706 (70.8)	233 (58.3)	473 (79.2)
≥ 5	291 (29.2)	167 (41.7)	124 (20.8)
Missing	*3*	*0*	*3*
**How much did the last client pay [in FCFA (USD)]**			
≤ 1999 (~3.50)	238 (23.8)	152 (38.0)	86 (14.3)
[2000 – 4999] (3.50 – 8.75)	287 (28.7)	138 (34.5)	149 (24.8)
[5000 – 9999] (8.75 – 17.50)	241 (24.1)	69 (17.2)	172 (28.7)
≥ 10000 (17.50)	234 (23.4)	41 (10.3)	193 (32.2)
**Ever suffered assault/coerced sexual intercourse**			
Yes	115 (11.7)	41 (10.5)	74 (12.6)
No	866 (88.3)	351 (89.5)	515 (87.4)
Missing	*19*	*8*	*11*

[1]Most FSWs meet clients in more than one location: the total is not equal to 100%.

High HIV exposure despite the use of condoms

Overall in the questionnaire, 79% of FSWs in San Pedro and 92% of FSWs in Abidjan reported consistent condom use with their clients (Table 2). However, the question about regular use of condoms could not fully capture actual condom use; there were several situations where FSWs had unprotected sexual intercourse. 23% would accept condomless sex for a large sum of money. This exposure to unprotected sex was reported as well by several FSWs during the qualitative interviews and was explained by the critical need for money.

"And when you look back at your week, you didn't even make 2000 francs. You begin to think about it. Ah! Honestly, I do accept [unprotected sex]." (FGD, San Pedro)

Several interviewed women also attested that violent clients had assaulted them and refused to use condoms.

"They brutalize us. Often, they don't wear any condom. They force us. Often even, young junkies, they can come upon us. And they assault us." (In-depth interview, San Pedro, 28 years old).

Moreover, 86% of FSWs in San Pedro and 89% of FSWs in Abidjan reported not systematically using condoms with their regular partner, even though only 10% and 21%, respectively, knew their partner's HIV status. This practice was reported during interviews as well, even though the women explained that they perceived a risk associated with condomless intercourse. During a focus group discussion that took place in a slum in San Pedro, above a bar where FSWs meet clients, interviewed women were discussing about their regular partners. One of them stated that her boyfriend asked her to not use any condoms to prove her trust.

"This guy, he tells you I'm faithful to you. I want us to have sex without condoms to show trust. That's why I think that the scary person is your boyfriend, not the client." (FGD, San Pedro)

Another one explained the lack of trust in her partners was balanced by the fact that they would protect her from violent clients.

"Love is the only weapon where you sleep with your enemy (laughs). I mean, he's your closest enemy. He's the one who can kill you because he's not with you only. But you say, he's my official. You need him because he protects you." (FGD, San Pedro)

It seemed that the women were not in a strong negotiation position, and accepted to have condomless sex despite knowing their partners had concomitant relationships.

For peer review only - http://bmjopen.bmj.com/site/about/guidelines.xhtml

For peer review only

Furthermore, 51% of FSWs in San Pedro and 43% of FSWS in Abidjan had received their last HIV test less than 6 months before. However, 7% in San Pedro and 15% in Abidjan had never been tested before the survey.

So, if condom use was high in general, most FSWs were still exposed to HIV: 59% had at least one instance of condomless intercourse over the previous week. FSWs' responses to the first question assessing condom use might refer to "typical use" as opposed to specific circumstances.

During qualitative interviews, we presented PrEP as a medicine that could protect them against HIV if properly taken and would imply to have a regular medical follow-up. PrEP was not yet available in Côte d'Ivoire at the time of the interviews and women had never heard about it. However, several questions emerged in relation with concrete matters such as side-effects, cost, current availability in pharmacies, compatibility with pregnancy, appropriate reaction if one or more pills are forgotten, etc. Many interviewed women considered PrEP as useful to prevent HIV transmission from their partners in particular, as they felt obligated to not use any condoms with them.

"*Danger itself, it comes from the one beside me. That pill is welcome, because by taking it I protect myself against the one beside me*." (FGD, San Pedro)

PrEP was presented similarly, although more briefly, in the questionnaire. The large majority of surveyed FSWs (98.6%) showed interest in a medicine that could provide efficient protection against HIV, even if it required a medical follow-up every three months (99.4%).

Table 2. Use of condoms and HIV exposure in the quantitative survey

Variables	All women n (%) n=1000	San Pedro n (%) n=400	Abidjan n (%) n=600
**Condom use with clients**			
Never	9 (0.9)	3 (0.8)	6 (1.0)
Sometimes	29 (3.0)	17 (4.4)	12 (2.1)
Often	86 (8.9)	58 (15.1)	28 (4.8)
Always	837 (87.0)	304 (79.2)	533 (92.1)
Does not know/does not want to answer	2 (0.1)	2 (0.5)	0 (0.0)
Missing	*37*	*16*	*21*
**Use of condom with boyfriend/husband**			
Never	370 (53.2)	157 (50.5)	213 (55.3)
Sometimes	115 (16.5)	61 (19.6)	54 (14.0)
Often	123 (17.7)	49 (15.8)	74 (19.2)
Always	62 (8.9)	19 (6.1)	43 (11.2)
Does not know/does not want to answer	26 (3.8)	25 (8.0)	1 (0.3)
Missing	*18*	*6*	*12*
**Acceptance of condomless sexual intercourse in exchange for a large sum of money**			
Never	764 (77.4)	251 (63.9)	513 (86.4)
Sometimes	79 (8.0)	52 (13.2)	27 (4.5)
Often	92 (9.3)	55 (14.0)	37 (6.2)
Always	12 (1.2)	6 (1.5)	6 (1.0)
Does not know/does not want to answer	40 (4.0)	29 (7.4)	11 (1.9)
Missing	*13*	*7*	*6*
**At least one instance of condomless intercourse over last 7 days[1]**			
Yes	220 (58.8)	152 (72.0)	68 (41.5)
No	154 (41.1)	59 (28.0)	95 (57.9)
Does not want to answer	1 (0.2)	0 (0.0)	1 (0.6)
Missing	*625*	*189*	*436*
**Last HIV test (months)**			
< 6	458 (45.9)	230 (50.9)	255 (42.6)
[6 – 12[	239 (24.0)	98 (24.6)	141 (23.6)
≥ 12	182 (18.2)	69 (17.3)	113 (18.9)
Never	114 (11.4)	26 (6.5)	88 (14.7)
Does not know/does not want to answer	4 (0.4)	3 (0.7)	1 (0.2)

For peer review only - http://bmjopen.bmj.com/site/about/guidelines.xhtml

Missing	*3*	*1*	*2*
**Knowledge of boyfriend/husband's HIV status**			
Yes	121 (17.4)	33 (10.6)	88 (23.0)
No	573 (82.3)	279 (89.1)	294 (768)
Does not want to answer	2 (0.3)	1 (0.3)	1 (0.3)
Missing	*18*	*4*	*14*
**Interest in a medicine protecting against HIV**			
Yes	982 (98.6)	394 (99.0)	588 (98.3)
No	11 (1.1)	2 (0.5)	9 (1.5)
Does not know	3 (0.3)	2 (0.5)	1 (0.2)
Missing	*4*	*2*	*2*
**If yes, would agree to a medical follow-up every 3 months**			
Yes	964 (99.4)	391 (99.7)	573 (99.1)
No	5 (0.5)	1 (0.3)	4 (0.7)
Does not know	1 (0.1)	0 (0.0)	1 (0.2)
Missing	*12*	*2*	*10*

[1]This variable was added during the survey; for this reason, some participants did not answer the question.

For peer review only

Beyond HIV, many unmet SRH needs exist

In total, 43% of the survey participants had at least one unwanted pregnancy, and 50% had at least one abortion in their lifetime (Table 3). Only 39% of surveyed FSWs were using a contraceptive method other than condoms; among them, most FSWs in Abidjan mentioned taking the pill (70%) compared to only 33% of FSWs in San Pedro, where 35% declared using an implant. Unfortunately, child desire was not asked in the quantitative survey, which does not allow us to calculate the unmet need for contraception among FSWs. However, as a proxy, it appeared that most of the interviewed FSWs did not want a child at the moment.

"*My main risk, it is to not get pregnant because I'm still a schoolgirl. If I get pregnant, who will take care of it* [the baby]*?*" (In-depth interview, Abidjan, 18 years old)

However, women explained during interviews that they feared becoming sterile because of contraceptive means, especially the pill.

"They [peer educators from the community-based NGO] told me about the pill, but I refused because I don't have children yet. I don't want to have problems in the future." (In-depth interview, Abidjan, 18 years old)

"*That's what makes me tired. I'm afraid because I don't have children yet. That's my problem, otherwise for diseases, well, there are condoms.*" (In-depth interview, San Pedro, 19 years old)

Moreover, only half of the survey participants knew about emergency contraception, among which 36% knew only nonmedical means.

Additionally, 36% of survey participants practiced sex work during menstruation, mainly using tampons (62%) or cold water (24%) to stop the bleeding.

Finally, 79% of FSWs in San Pedro and 55% of FSWs in Abidjan reported contracting an STI over the past 12 months. Even though half of the questionnaire survey participants thought they were very exposed to HIV infection, the interviewed FSWs often declared being preoccupied by other diseases as well, such as STIs or cancer.

"But we, every time, when we go in the bush, it's not only AIDS that kills. There are several diseases. Today we talk about cervical cancer. So I think it's not only AIDS we should get protected from. We have to protect ourselves from many diseases that are sexual." (FGD, San Pedro)

Despite the work of peer educators at prostitution sites, few surveyed FSWs visited the dedicated community clinics, with 76% in San Pedro and 61% in Abidjan consulting a health practitioner over the past year, among which 40% in San Pedro and 17% in Abidjan went to a dedicated facility. In interviews,

For peer review only - http://bmjopen.bmj.com/site/about/guidelines.xhtml

some FSWs reported the inconvenient opening times and/or location or the fear of being identified as an FSW in the clinic area as reasons for not visiting these clinics. In the event of condom breakage, FSWs usually relied on self-medication. For example, a young woman described the beverages she would use for a vaginal douche.

"*I'm going to buy Coke with Nescafe. It's for cleaning everything falling down.*" (In-depth interview, Abidjan, 17 years old)

They act similarly in case of suspicion of an STI.

"*Before going to the clinic, we try traditional plants and medicines first*." (FGD, San Pedro)

Table 3. Sexual and reproductive health in the quantitative survey

Variables	All women n (%) n=1000	San Pedro n (%) n=400	Abidjan n (%) n=600
**Had at least one undesired pregnancy**			
Yes	416 (42.9)	173 (45.1)	243 (41.5)
No	554 (57.1)	211 (54.9)	343 (58.5)
Missing	*30*	*16*	*14*
**Had at least one abortion**			
Yes	488 (50.2)	195 (50.4)	293 (50.0)
No	485 (49.8)	192 (49.6)	293 (50.0)
Missing	*27*	*13*	*14*
**Use of contraception other than condom**			
Yes	391 (39.1)	193 (48.3)	198 (33.0)
No	608 (60.8)	206 (51.5)	402 (67.0)
Does not know	1 (0.1)	1 (0.2)	0 (0.0)
**If yes, which contraceptive method[2]**			
Pill	204 (52.0)	65 (33.5)	139 (70.2)
Injectable	91 (23.3)	55 (28.5)	36 (18.2)
Implant	83 (21.2)	68 (35.1)	15 (7.6)
Other method[1] (nivaquine, spice, traditional medicine, etc.)	24 (6.1)	13 (6.7)	11 (5.6)
**Knowledge of emergency contraception**			
Yes	472 (48.4)	195 (50.8)	277 (46.9)
No	497 (51.0)	183 (47.7)	314 (53.1)
Does not know	6 (0.6)	6 (1.5)	0 (0.0)
Missing	*25*	*16*	*9*
**If yes, type of emergency contraception known[2]**			
Morning-after pill	304 (64.1)	100 (49.7)	204 (74.7)
Other[1] (antibiotic, coffee, soda, salted water, and lemon)	170 (35.9)	101 (50.3)	69 (25.3)
**Sex work during menstruation**			
Yes	363 (36.4)	154 (38.5)	209 (34.9)
No	635 (63.6)	246 (61.5)	389 (65.1)
Missing	*2*	*0*	*2*
**If yes, tool used for sex work during menstruation[2]**			
Wash with ice-cold water	86 (24.0)	37 (24.2)	49 (23.7)
Piece of ice	26 (7.3)	11 (7.2)	15 (7.3)
Tampon	222 (62.0)	105 (68.6)	117 (57.1)
Other tools[1] (hot water, soapy water, cotton, etc.)	49 (13.7)	12 (7.8)	37 (17.9)
**Self-reported STI (last 12 months)**			
Yes	639 (64.7)	312 (78.8)	327 (55.2)
No	349 (35.3)	84 (21.2)	265 (44.8)
Missing	*12*	*4*	*8*
**Last medical consultation**			
Less than 3 months	195 (19.6)	101 (25.4)	94 (15.7)
3 – 12 months	475 (47.7)	202 (50.8)	273 (45.6)
More than a year	258 (25.9)	79 (19.8)	179 (29.9)
Never consulted	68 (6.8)	16 (4.0)	52 (8.7)

For peer review only

Missing	*4*	*2*	*2*
**If ever consulted, site of the last consultation with a doctor/nurse**			
Dedicated facility	225 (26.3)	141 (40.5)	84 (16.6)
Public facility	415 (48.6)	123 (35.3)	292 (57.7)
Private facility	213 (24.9)	83 (23.8)	130 (25.7)
Does not know	1 (0.1)	1 (0.3)	0 (0.0)
Missing	*74*	*34*	*40*

[1]"Other" categories describe participants' specific answers.
[2]Several possible answers.

DISCUSSION

Both the quantitative and qualitative results showed that FSWs were highly exposed to HIV despite their use of condoms. There were in fact a variety of situations in which the surveyed FSWs had condomless sex. First, the large majority did not use condoms with their regular partner despite their acknowledged concurrent sexual partnerships. They experienced coercion on the part of their male partners, questioning their faith in the relationship. Having condomless sex is a proof of trust that is difficult to negotiate, and women experience low decision-making power when facing the primacy of men's sexual pleasure (17), in a context where gender norms reinforce male domination over women (18). Second, some FSWs accepted condomless sexual intercourse for a large sum of money, especially when they had had few previous clients. Financial need associated with low prices of sexual intercourses and irregular weekly earnings drove some FSWs to engage in condomless sex as a way to earn more. Third, the violence or the threat thereof that FSWs faced sometimes prevented them to negotiate condom use. Different studies showed that women who are victims of abuse are less likely to use condoms with their clients than those who are not (19,20). It has been suggested that sexual and physical assaults from clients can lead to condom breakage (21). Performing an illegal activity can also compel women, especially in the street, to negotiate quickly with clients at the expense of condom use. The situation appeared even worse in some rural areas around San Pedro, where the interviewed FSWs revealed that they could not buy any condoms in the village, as there was no point of sale. According to WHO, the main criteria to implement PrEP in a population is the high incidence of HIV in that population (6). Thus, in a complementary study within the PrEP-CI project (not yet published), we calculated the incidence among the 1000 surveyed FSWs: through a recent infection testing algorithm adapted to the Ivorian context, we found that the incidence among surveyed FSWs was 2.2% (1.5% in Abidjan and 3.2% in San Pedro) (16). In this context, it appears that PrEP could be an appropriate tool for preventing HIV in this population.

The majority of interviewed and surveyed FSWs had low awareness and knowledge of PrEP before our study but most of them were highly willing to use this medicine despite the constraint of regular medical follow-up. Participants felt PrEP would give added protection against infection, in particular with regular partners. A study in Kenya had similar findings and suggested to promote PrEP through outreach activities for sex workers (22). However, a PrEP implementation trial in South Africa showed low adherence despite high declared acceptability before the implementation (14). For this reason we were attentive to challenges that might hinder PrEP uptake and adherence for FSWs.

Our results showed that FSWs faced many unmet needs regarding SRH beyond HIV prevention and treatment. Inconsistent condom use exposed FSWs to STIs (23,24) and undesired pregnancies (25), which could increase their mortality and morbidity (26). The prevalence of contraceptive use was low in the surveyed population despite their high risk of undesired pregnancy due to the common fear of contraception causing sterility (27). Furthermore, using ice or tissues to continue sex work during menstruation has been proven to be a source of bacterial infections (28). These needs could be

For peer review only - http://bmjopen.bmj.com/site/about/guidelines.xhtml

addressed in the community clinics of the two NGOs. However, as shown in other studies, FSWs faced many obstacles in accessing SRH care, because of the high costs or distance of the sites (29), the stigmatizing and discriminating attitudes of some health practitioners, the FSWs' social and economic marginalization, and restrictive laws related to their activity (30). FSWs thus preferred advice from their peers or self-medication. Moreover, peer educators from both NGOs expressed that public policies and international donors in Côte d'Ivoire currently focus on the identification and referral of new cases of HIV-positive FSWs, while HIV-negative women have limited access to care as stated above. A PrEP program requires a medical follow-up every three months and thus implies to consider the chronic follow-up of HIV-negative women. Integrating SRH services (such as contraception or STI testing and treatment) into a PrEP program could be a way to attract them into care and follow-up, as surveyed and interviewed FSWs expressed concerns about other diseases and health needs than just HIV prevention and care.

In addition, FSWs in San Pedro appeared to be in a more precarious situation than those in Abidjan due to their lower education level, higher number of children, irregularity of work, multiplicity of clients and work locations, and the lower price of sexual intercourse. They were also more likely to have condomless intercourse, notably for a large sum of money, and to report having had an STI over the past year. This can be explained by the fact that a large percentage of FSWs in San Pedro came to the area during the period of coffee and cocoa exploitation (September-December), which brought many migrant workers; this results in less stability and security. The high mobility of these women generates "seasons of risk" (31), i.e., times when an individual might face an increased risk of HIV infection. It is paramount to take this into account when implementing daily PrEP for these women (32); they are the population most likely to regularly suspend their PrEP use. Developing mobile clinics that deliver HIV and SRH care services directly at prostitution sites could mitigate the issue of FSWs' mobility and address barriers in access to care, such as distance between prostitution sites and clinics or stigmatization associated with their activity.

FSWs' needs for PrEP cannot be understood without additionally considering the broader contexts in which their risk of exposure to HIV is situated: the context of their work, their relationships and their concerns about family planning or stigmatization, etc. This is important information to consider if a PrEP program is to successfully serve this at-risk population. In order to address women's experiences and concerns, a global SRH care package delivered through both community clinics and mobile clinics on prostitution sites appear essential. Several studies related to PrEP and SRH needs of FSWs showed as well that combination prevention approaches are necessary. First, as pointed by a study in Zimbabwe (33), women need to perceive the risk of getting infected by HIV and to be able to access health services in order to take PrEP daily. Second, as shown by Dhana *et al.* in a systematic review (34), there is a lack of coordination between SRH and HIV services dedicated to FSWs in Africa; service delivery models should integrate SRH services. Our results bring two considerations. First, in order to minimize stigma related to entry into care, services for HIV-positive and services for HIV-negative should not be dissociated. Second, rather than a PrEP program with additional services, a paradigm shift toward a patient-focused approach is needed, offering SRH services in which PrEP is an option but not mandatory. Not taking these issues into account might result in low adherence and impact the efficacy of a PrEP program.

A strength of this study is the use of mixed-method allowing us to better describe and understand challenges of PrEP implementation among FSWs in Côte d'Ivoire, as well as the strong collaboration with two NGOs helping us to reach the FSWs. Yet, this study has some limitations. First, as it focused on FSWs reached by two NGOs, the included population was probably more likely to know about HIV prevention and to access SRH care. In addition, our sample did not include occasional or undeclared

For peer review only - http://bmjopen.bmj.com/site/about/guidelines.xhtml

FSWs. As such, the results cannot be extrapolated to all FSWs working inside or around Abidjan and San Pedro but can provide an operational perspective for developing healthcare services. A matter of concern, pointed out by field workers and data collected, are the young underage FSWs (three interviews were conducted with FSWs aged less than 18 on the beaches of Abidjan). Ivorian law authorizes HIV testing for teenagers aged 16 and 17, without parental consent. What about other care and services that cannot be delivered to them in the absence of consent?

CONCLUSIONS

Implementing PrEP among FSWs in West Africa, such as in Côte d'Ivoire, is not only about providing a new prevention tool but is also an invitation to consider the chronic follow-up of HIV-negative FSWs. A global care package should be offered to FSWs, including HIV prevention and care, STI screening and treatment, contraception, menstrual management counseling and HBV screening, vaccination and medical treatment. In addition, PrEP initiation should not limit access to SRH services; conversely, SRH services could be a way to attract FSWs to HIV prevention. Beyond reducing the risk of HIV among FSWs and their partners, PrEP provides an opportunity to improve their health condition more globally.

While current policies focus on only HIV-infected women and on the importance of testing new FSWs, our results highlight the importance of developing a people-focused approach, as opposed to an "HIV-focused approach", that integrates all SRH needs when transitioning from PrEP efficacy trials to implementation (15).

6. Conflict of interest statement

There are no conflicts of interest.

7. Author Statement

JL, SE and CD designed the ANRS 12361 PrEP-CI study. JMM and MN implemented the quantitative survey with the support of CA and SK. VB conducted the qualitative interviews. VB and JL developed the research question addressed in this paper. VB did the qualitative analysis, and MN did the statistical analysis. VB wrote the manuscript with the support of JL, MP and MN. All authors contributed to the interpretation and presentation of the findings. All authors approved the final version of the manuscript for submission.

8. Funding

The PrEP-CI ANRS 12361 was funded by the Bill and Melinda Gates Foundation (Investment ID: OPP1106343) and the French National Agency for AIDS and Viral Hepatitis Research (ANRS).

9. Acknowledgments

We would like to thank all participants as well as Aprosam's and Espace Confiance's peer educators.

10. Data sharing statement

Dataset are available upon request on Zenodo.

11. References

1. Molina J-M, Capitant C, Spire B, Pialoux G, Cotte L, Charreau I, et al. On-Demand Preexposure Prophylaxis in Men at High Risk for HIV-1 Infection. New England Journal of Medicine. 2015 Dec 3;373(23):2237–46.

For peer review only - http://bmjopen.bmj.com/site/about/guidelines.xhtml

2. Grant RM, Lama JR, Anderson PL, McMahan V, Liu AY, Vargas L, et al. Preexposure chemoprophylaxis for HIV prevention in men who have sex with men. N Engl J Med. 2010 Dec 30;363(27):2587–99.
3. Baeten JM, Donnell D, Ndase P, Mugo NR, Campbell JD, Wangisi J, et al. Antiretroviral Prophylaxis for HIV Prevention in Heterosexual Men and Women. New England Journal of Medicine. 2012 Aug 2;367(5):399–410.
4. Choopanya K, Martin M, Suntharasamai P, Sangkum U, Mock PA, Leethochawalit M, et al. Antiretroviral prophylaxis for HIV infection in injecting drug users in Bangkok, Thailand (the Bangkok Tenofovir Study): a randomised, double-blind, placebo-controlled phase 3 trial. Lancet. 2013 Jun 15;381(9883):2083–90.
5. Thigpen MC, Kebaabetswe PM, Paxton LA, Smith DK, Rose CE, Segolodi TM, et al. Antiretroviral Preexposure Prophylaxis for Heterosexual HIV Transmission in Botswana. New England Journal of Medicine. 2012 Aug 2;367(5):423–34.
6. WHO. Guideline on when to start antiretroviral therapy and on pre-exposure prophylaxis for HIV [Internet]. Geneva: World Health Organisation; 2015 Sep p. 78. Report No.: 978 92 4 150956 5. Available from: http://apps.who.int/iris/bitstream/10665/186275/1/9789241509565_eng.pdf
7. UNAIDS. UNAIDS data 2017 [Internet]. UNAIDS; 2017 [cited 2018 Jul 11]. Available from: http://www.unaids.org/sites/default/files/media_asset/2017_data-book_en.pdf
8. UNAIDS. On the Fast-Track to end AIDS by 2030: Focus on location and population [Internet]. 2015 p. 268. Available from: http://www.unaids.org/en/resources/documents/2015/FocusLocationPopulation
9. Corneli AL, Deese J, Wang M, Taylor D, Ahmed K, Agot K, et al. FEM-PrEP: Adherence Patterns and Factors Associated With Adherence to a Daily Oral Study Product for Pre-exposure Prophylaxis. J Acquir Immune Defic Syndr. 2014 Jul 1;66(3):324–31.
10. Marrazzo JM, Ramjee G, Richardson BA, Gomez K, Mgodi N, Nair G, et al. Tenofovir-based preexposure prophylaxis for HIV infection among African women. N Engl J Med. 2015 Feb 5;372(6):509–18.
11. Béhanzin L, Guédou FA, Geraldo N, Goma Matsétsé E, Aza-Gnandji M, Imorou Bah Chabi A, et al. PrEP and Early Antiretroviral Therapy Demonstration Project: Challenges to Ensure Follow-up and Adherence Among Female Sex Workers in Cotonou, Benin. In Chicago: HIVR4P 2016 Conference; 2016.
12. Alary M, Béhanzin L, Mboup A, Guédou FA, Geraldo N, Goma-Matsétsé E, et al. Early Antiretroviral Therapy and Daily Pre-exposure Prophylaxis for HIV Prevention Among Female Sex Workers in Cotonou, Benin: A Demonstration Study. In Madrid: HIVR4P 2016 Conference; 2018.
13. Eakle R, Gomez GB, Naicker N, Bothma R, Mbogua J, Cabrera Escobar MA, et al. HIV pre-exposure prophylaxis and early antiretroviral treatment among female sex workers in South Africa: Results from a prospective observational demonstration project. PLoS Med. 2017;14(11):1–17.
14. Eakle R, Bourne A, Mbogua J, Mutanha N, Rees H. Exploring acceptability of oral PrEP prior to implementation among female sex workers in South Africa. J Intern AIDS Soc. 2018 Feb 1;21(2):n/a-n/a.
15. Larmarange J, Becquet V, Masumbuko J-M, Nouaman M, Plazy M, Danel C, et al. Implementing preexposure prophylaxis among key populations: an opportunity for patient-centered services and management of hepatitis B. AIDS. 2018 Mar 27;32(6):829–30.
16. Nouaman MN, Becquet V, Masumbuko J-M, Anoma C, Soh K, Plazy M, et al. Évaluation de l’incidence du VIH chez des travailleuses du sexe en Côte d’Ivoire (PrEP-CI ANRS 12361). In Bordeaux; 2018.
17. Ghimire L, Smith WCS, van Teijlingen ER, Dahal R, Luitel NP. Reasons for non- use of condoms and self- efficacy among female sex workers: a qualitative study in Nepal. BMC Women’s Health [Internet]. 2011 Sep 26;11(42). Available from: https://doi.org/10.1186/1472-6874-11-42
18. Deering KN, Shaw SY, Thompson LH, Ramanaik S, Raghavendra T, Doddamane M, et al. Fertility intentions, power relations and condom use within intimate and other non-paying partnerships of women in sex work in Bagalkot District, South India. AIDS Care. 2015 Oct 3;27(10):1241–9.

For peer review only - http://bmjopen.bmj.com/site/about/guidelines.xhtml

19. Wirtz AL, Schwartz S, Ketende S, Anato S, Nadedjo FD, Ouedraogo HG, et al. Sexual Violence, Condom Negotiation, and Condom Use in the Context of Sex Work: Results From Two West African Countries. JAIDS Journal of Acquired Immune Deficiency Syndromes. 2015 Mar;68:S171.
20. Decker MR, McCauley HL, Phuengsamran D, Janyam S, Seage GR, Silverman JG. Violence victimisation, sexual risk and sexually transmitted infection symptoms among female sex workers in Thailand. Sexually Transmitted Infections. 2010 Jun 1;86(3):236–40.
21. Choi SYP, Chen KL, Jiang ZQ. Client-Perpetuated Violence and Condom Failure Among Female Sex Workers in Southwestern China. Sexually Transmitted Diseases. 2008 Feb;35(2):141.
22. Restar AJ, Tocco JU, Mantell JE, Lafort Y, Gichangi P, Masvawure TB, et al. Perspectives on HIV Pre- and Post- Exposure Prophylaxes (PrEP and PEP) among Female and Male Sex Workers in Mombasa, Kenya: Implications for Integrating Biomedical Prevention into Sexual Health Services. AIDS Educ Prev. 2017 Apr;29(2):141–53.
23. Braunstein SL, Ingabire CM, Kestelyn E, Uwizera AU, Mwamarangwe L, Ntirushwa J, et al. High Human Immunodeficiency Virus Incidence in a Cohort of Rwandan Female Sex Workers. Sexually Transmitted Diseases. 2011 May;38(5):385.
24. Scorgie F, Chersich MF, Ntaganira I, Gerbase A, Lule F, Lo Y-R. Socio-Demographic Characteristics and Behavioral Risk Factors of Female Sex Workers in Sub-Saharan Africa: A Systematic Review. AIDS Behav. 2012 May 1;16(4):920–33.
25. Schwartz S, Papworth E, Thiam-Niangoin M, Abo K, Drame F, Diouf D, et al. An urgent need for integration of family planning services into HIV care: the high burden of unplanned pregnancy, termination of pregnancy, and limited contraception use among female sex workers in Côte d'Ivoire. JAIDS Journal of Acquired Immune Deficiency Syndromes. 2015 Mar 1;68(Supplement 3):S91–S98.
26. Mullick S. Sexually transmitted infections in pregnancy: prevalence, impact on pregnancy outcomes, and approach to treatment in developing countries. Sexually Transmitted Infections. 2005 Aug 1;81(4):294–302.
27. Guillaume A, Ltd. A. The Role of Abortion in the Fertility Transition in Abidjan (Côte d'Ivoire) during the 1990s. Population (English Edition, 2002-). 2003;58(6):657–85.
28. Baisley K, Changalucha J, Weiss HA, Mugeye K, Everett D, Hambleton I, et al. Bacterial vaginosis in female facility workers in north-western Tanzania: prevalence and risk factors. Sexually Transmitted Infections. 2009 Sep 1;85(5):370–5.
29. Wahed T, Alam A, Sultana S, Rahman M, Alam N, Martens M, et al. Barriers to sexual and reproductive healthcare services as experienced by female sex workers and service providers in Dhaka city, Bangladesh. PLoS ONE. 2017 31;12(7).
30. Ippoliti NB, Nanda G, Wilcher R. Meeting the Reproductive Health Needs of Female Key Populations Affected by HIV in Low-and Middle-Income Countries: A Review of the Evidence. Studies in Family Planning. 2017;48(2):121–151.
31. Elsesser SA, Oldenburg CE, Biello KB, Mimiaga MJ, Safren SA, Egan JE, et al. Seasons of Risk: Anticipated Behavior on Vacation and Interest in Episodic Antiretroviral Pre-exposure Prophylaxis (PrEP) Among a Large National Sample of U.S. Men Who have Sex with Men (MSM). AIDS Behav. 2016 Jul;20(7):1400–7.
32. Namey E, Agot K, Ahmed K, Odhiambo J, Skhosana J, Guest G, et al. When and why women might suspend PrEP use according to perceived seasons of risk: implications for PrEP-specific risk-reduction counselling. Culture, Health & Sexuality. 2016 Sep;18(9):1081–91.
33. Cowan FM, Delany-Moretlwe S. Promise and pitfalls of pre-exposure prophylaxis for female sex workers. Curr Opin HIV AIDS. 2016 Jan;11(1):27–34.
34. Dhana A, Luchters S, Moore L, Lafort Y, Roy A, Scorgie F, et al. Systematic review of facility-based sexual and reproductive health services for female sex workers in Africa. Globalization and Health. 2014 Jun 10;10:46.

For peer review only - http://bmjopen.bmj.com/site/about/guidelines.xhtml

STROBE Statement—Checklist of items that should be included in reports of ***cross-sectional studies***

	Item No	Recommendation	Reported on page #
**Title and abstract**	1	(*a*) Indicate the study's design with a commonly used term in the title or the abstract	1
		(*b*) Provide in the abstract an informative and balanced summary of what was done and what was found	2
**Introduction**			
Background/rationale	2	Explain the scientific background and rationale for the investigation being reported	3
Objectives	3	State specific objectives, including any prespecified hypotheses	3
**Methods**			
Study design	4	Present key elements of study design early in the paper	3
Setting	5	Describe the setting, locations, and relevant dates, including periods of recruitment, exposure, follow-up, and data collection	3-4
Participants	6	(*a*) Give the eligibility criteria, and the sources and methods of selection of participants	4
Variables	7	Clearly define all outcomes, exposures, predictors, potential confounders, and effect modifiers. Give diagnostic criteria, if applicable	4-5
Data sources/ measurement	8*	For each variable of interest, give sources of data and details of methods of assessment (measurement). Describe comparability of assessment methods if there is more than one group	5
Bias	9	Describe any efforts to address potential sources of bias	
Study size	10	Explain how the study size was arrived at	6
Quantitative variables	11	Explain how quantitative variables were handled in the analyses. If applicable, describe which groupings were chosen and why	5
Statistical methods	12	(*a*) Describe all statistical methods, including those used to control for confounding	5
		(*b*) Describe any methods used to examine subgroups and interactions	5
		(*c*) Explain how missing data were addressed	5
		(*d*) If applicable, describe analytical methods taking account of sampling strategy	
		(*e*) Describe any sensitivity analyses	
**Results**			
Participants	13*	(a) Report numbers of individuals at each stage of study—eg numbers potentially eligible, examined for eligibility, confirmed eligible, included in the study, completing follow-up, and analysed	6-7
		(b) Give reasons for non-participation at each stage	6-7
		(c) Consider use of a flow diagram	
Descriptive data	14*	(a) Give characteristics of study participants (eg demographic, clinical, social) and information on exposures and potential confounders	6-7
		(b) Indicate number of participants with missing data for each variable of interest	Cf. tables
Outcome data	15*	Report numbers of outcome events or summary measures	
Main results	16	(*a*) Give unadjusted estimates and, if applicable, confounder-adjusted estimates and their precision (eg, 95% confidence interval). Make clear which confounders were adjusted for and why they were included	Cf. tables

For peer review only - http://bmjopen.bmj.com/site/about/guidelines.xhtml

		(*b*) Report category boundaries when continuous variables were categorized	Cf. tables
		(*c*) If relevant, consider translating estimates of relative risk into absolute risk for a meaningful time period	
Other analyses	17	Report other analyses done—eg analyses of subgroups and interactions, and sensitivity analyses	
**Discussion**			
Key results	18	Summarise key results with reference to study objectives	10
Limitations	19	Discuss limitations of the study, taking into account sources of potential bias or imprecision. Discuss both direction and magnitude of any potential bias	11
Interpretation	20	Give a cautious overall interpretation of results considering objectives, limitations, multiplicity of analyses, results from similar studies, and other relevant evidence	10-11
Generalisability	21	Discuss the generalisability (external validity) of the study results	11
**Other information**			
Funding	22	Give the source of funding and the role of the funders for the present study and, if applicable, for the original study on which the present article is based	14

*Give information separately for exposed and unexposed groups.

Note: An Explanation and Elaboration article discusses each checklist item and gives methodological background and published examples of transparent reporting. The STROBE checklist is best used in conjunction with this article (freely available on the Web sites of PLoS Medicine at http://www.plosmedicine.org/, Annals of Internal Medicine at http://www.annals.org/, and Epidemiology at http://www.epidem.com/). Information on the STROBE Initiative is available at www.strobe-statement.org.

For peer review only

BMJ Open

Sexual health needs of female sex workers in Côte d'Ivoire: a mixed-methods study to prepare the future implementation of pre-exposure prophylaxis (PrEP) for HIV prevention

Journal:	*BMJ Open*
Manuscript ID	bmjopen-2018-028508.R1
Article Type:	Original research
Date Submitted by the Author:	01-Oct-2019
Complete List of Authors:	Becquet, Valentine; Centre Population et Développement, Institut de Recherche pour le Développement, Université Paris Descartes, ERL Inserm U 1244<br>Nouaman, Marcellin; Programme PAC-CI/ANRS Research Site<br>Plazy, Mélanie; Inserm Research Center 1219 (Bordeaux Population Health), ISPED, Université de Bordeaux<br>Masumbuko, Jean-Marie; Programme PAC-CI/ANRS Research Site<br>Anoma, Camille; Espace Confiance<br>Kouame, Soh; Aprosam<br>Danel, Christine; Programme PAC-CI/ANRS Research Site<br>Eholie, Serge; Programme PAC-CI/ANRS Research Site<br>Larmarange, Joseph; Centre Population et Développement, Institut de Recherche pour le Développement, Université Paris Descartes, ERL Inserm U 1244
**Primary Subject Heading**:	Sexual health
Secondary Subject Heading:	HIV/AIDS, Qualitative research
Keywords:	QUALITATIVE RESEARCH, HIV & AIDS < INFECTIOUS DISEASES, Public health < INFECTIOUS DISEASES, Demography < TROPICAL MEDICINE, Epidemiology < TROPICAL MEDICINE

SCHOLARONE™ Manuscripts

1. Title page

Sexual health needs of female sex workers in Côte d'Ivoire: a mixed-methods study to prepare the future implementation of pre-exposure prophylaxis (PrEP) for HIV prevention

Valentine Becquet[1§], Marcellin Nouaman[2], Mélanie Plazy[3], Jean-Marie Masumbuko[2], Camille Anoma[4], Soh Kouamé[5], Christine Danel[2], Serge Eholié[2], Joseph Larmarange[1] for the ANRS 12361 PrEP-CI Study group

[1]Centre Population et Développement, Institut de Recherche pour le Développement, Université Paris Descartes, ERL Inserm U 1244, Paris, France

[2]Programme PAC-CI site ANRS de Côte d'Ivoire, Abidjan, Côte d'Ivoire

[3]Inserm Research Center 1219 (Bordeaux Population Health), ISPED, Université de Bordeaux, Bordeaux, France

[4]Espace Confiance, Abidjan, Côte d'Ivoire

[5]Aprosam, San Pedro, Côte d'Ivoire

§ Correspondance to Valentine Becquet, PhD, Centre Population et Développement, Université Paris Descartes, 45 rue des Saints-Pères, 75006 Paris, France. +33660082287

Email addresses:
VB: valentine.becquet@gmail.com
MN: nouaman_et_vie@yahoo.fr
MP: melanie.plazy@u-bordeaux.fr
JMM: masumbukojm@gmail.com
CA: anomacamille@yahoo.fr
SK: sohkouame1@yahoo.fr
CD: christinemarie.danel@gmail.com
SE: sergeholie@yahoo.fr
JL: joseph.larmarange@ceped.org

Members of the ANRS 12361 PrEP-CI study group:

» ABOUBAKAR Sangaré (Aprosam, San Pedro, Côte d'Ivoire)
» ANGLARET Xavier (PAC-CI, Abidjan, Côte d'Ivoire / Inserm, Bordeaux, France)
» ANOMA Camille (Espace Confiance, Abidjan, Côte d'Ivoire)
» BARIN Francis (Université François Rabelais, Tours, France)
» BAZIN Brigitte (ANRS, Paris, France)
» BECQUET Valentine (Ceped/IRD, Paris, France)
» DABIS François (ISPED/Inserm, Bordeaux, France)
» DANEL Christine (PAC-CI, Abidjan, Côte d'Ivoire / Inserm, Bordeaux, France)
» EHOLIE Serge (PAC-CI, Abidjan, Côte d'Ivoire)
» EKOUEVI Didier (PAC-CI, Abidjan, Côte d'Ivoire)
» FONSART Julien (Hôpital Saint-Louis, Paris, France)
» GBOSI Kate (Aprosam, San Pedro, Côte d'Ivoire)
» KWAMÉ Abo (Programme National de Lutte contre le Sida, Côte d'Ivoire)
» LARMARANGE Joseph (Ceped/IRD, Paris, France)
» MASUMBUKO Jean-Marie (PAC-CI, Abidjan, Côte d'Ivoire)

For peer review only

For peer review only - http://bmjopen.bmj.com/site/about/guidelines.xhtml

» MÉDA Nicolas (Centre Muraz, Bobo-Dioulasso, Burkina Faso)
» MOH Raoul (PAC-CI, Abidjan, Côte d'Ivoire)
» MOLINA Jean-Michel (Hôpital Saint-Louis, Paris, France)
» N'DRI-YOMAN Thérèse (PAC-CI, Abidjan, Côte d'Ivoire)
» NOUAMAN Marcellin (PAC-CI, Abidjan, Côte d'Ivoire)
» PLAZY Mélanie (ISPED / Inserm, Bordeaux, France)
» SOH Kouamé (Aprosam, San Pedro, Côte d'Ivoire)
» TANOE Solange (Espace Confiance, Abidjan, Côte d'Ivoire)
» YEO Roselyne (Espace Confiance, Abidjan, Côte d'Ivoire)

For peer review only

Word count : 4912

For peer review only - http://bmjopen.bmj.com/site/about/guidelines.xhtml

2. Keywords (6)

HIV prevention; sexual and reproductive health; people-focused approach; pre-exposure prophylaxis (PrEP); sex work; mixed-methods research

3. Abstract

Objective

Describe sexual and reproductive health (SRH) needs of female sex workers (FSWs) to inform the future implementation of pre-exposure prophylaxis (PrEP) for HIV prevention in this population.

Design and setting

The ANRS 12361 PrEP-CI cross-sectional and mixed-methods study was designed and implemented with two community-based organizations in Côte d'Ivoire.

Participants

A convenience sample of 1000 FSWs aged ≥18, not known as HIV+, completed a standardized questionnaire assessing sociodemographic characteristics, sexual practices and behaviors, use of community health services, *a priori* acceptability of PrEP.

Twenty-two in-depth interviews and eight focus-group discussions were also conducted to document FSWs' risky practices and sexual behaviors, experiences with violence and discrimination, attitudes regarding HIV and sexually transmitted infections (STIs), and barriers to SRH services.

Results

Although 87% of FSWs described consistent condom use with clients, >22% declared accepting condomless sexual intercourse for a large sum of money. Furthermore, condom use with their steady partner and knowledge of their partner's HIV status was low, despite their acknowledged concurrent sexual partnerships.

While inconsistent condom use exposed FSWs to STIs and undesired pregnancies, the prevalence of contraceptive use was low (39%) due to fear of contraception causing sterility. FSWs faced obstacles in accessing SRH care and preferred advice from their peers or self-medication.

Conclusions

Despite adoption of preventive behavior in most cases, FSWs are still highly exposed to HIV. Furthermore, FSWs seem to face several barriers in accessing SRH. Implementing PrEP among FSWs in West Africa, such as in Côte d'Ivoire, constitutes an opportunity to consider the chronic follow-up of HIV-negative FSWs. PrEP initiation should not condition access to SRH services; conversely, SRH services could be a way to attract FSWs into HIV prevention. Our results highlight the importance of developing a people-focused approach that integrates all SRH needs when transitioning from PrEP efficacy trials to implementation.

4. Article Summary

Strengths and limitations of this study

- Combination of a quantitative survey to document sexual and health behaviors and needs of FSWs and of a qualitative survey to understand rationales behind these behaviors and needs
- Use of a convenience sample representative of FSWs actually reached by two community NGOs but not representative of the overall population of FSWs
- A comprehensive descriptive study but not powered enough for multivariate explicative analysis

For peer review only

For peer review only - http://bmjopen.bmj.com/site/about/guidelines.xhtml

5. Main text

INTRODUCTION

Despite global progress in reducing new HIV infections and AIDS-related deaths in the last 10 years in sub-Saharan Africa (1,2), current policies and programmes are focusing on the identification of HIV-infected people in order to link them to HIV care and treatment (3,4), knowing that antiretroviral treatment has been proven to reduce HIV transmission (5). However, the number of new HIV infections still remain too high to achieve epidemic control (6). Numerous trials have shown that oral preexposure prophylaxis (PrEP), when taken correctly, was highly efficacious to prevent HIV acquisition, in particular among men having sex with men (MSM) (7–9). Since 2015, oral PrEP has been recommended by the World Health Organisation (WHO) for population at "substantial risk" of HIV acquisition (10).

In West Africa, most countries have mixed HIV epidemics, with a relatively low prevalence in the general population (compared to that in Eastern and Southern Africa) but severely affected key populations, particularly female sex workers (FSWs) and MSM (11). In Côte d'Ivoire in 2012, the HIV prevalence was estimated to be 29% in FSWs (12) and 19% among MSM (12). The National Program against HIV/AIDS (PNLS) requested operational research and data on the relevance of PrEP in order to better consider its future integration in the national algorithm of HIV prevention. A PrEP demonstration project (ANRS 12324 CohMSM) is currently being implemented among MSM. Our research team was invited to explore the sexual health needs of FSWs in this country as a potential target for a future PrEP program, knowing that PrEP was not yet available.

Although PrEP is effective when taken properly, the FEM-PREP (13) and VOICE (14) trials conducted among women from the general population in Southern and Eastern Africa showed low adherence to the treatment, resulting in a low or even null effect of PrEP. Similarly, PrEP implementation trials conducted among FSWs in Africa showed varying results regarding retention. In Benin, the retention rate after 10 months was 66% (15) and the overall retention rate after a complete follow-up of 28 months was 48% (16). In South Africa, it was 22% after 12 months (17) despite a high declared acceptability of PrEP before the implementation (18). Moreover, PrEP constitutes a new HIV prevention tool but does not prevent sexually transmitted infections (STIs) or unwanted pregnancies. It is therefore necessary to consider the overall needs of target populations in terms of sexual and reproductive health (SRH) (19). More operational and social science research is needed for the implementation of PrEP to be a success, especially on creating demand for oral PrEP; improving adherence; understanding the social and behavioral impact of PrEP; and integrating PrEP services with other services (10).

In this context, in order to design a future PrEP program targeting FSWs, the ANRS 12361 PrEP-CI pilot study was implemented to explore sexual health care needs that should be considered within such a program and to better describe FSWs currently reached by peer educators. This paper aims to describe the work and social environment of FSWs, their SRH needs and possible barriers for accessing care in two different settings in Côte d'Ivoire; i.e. different elements that need to be taken into account when implementing PrEP. We adopted a mixed approach: a quantitative survey was used to reach a high number of FSWs in order to be able to calculate incidence of HIV infection and to compare sexual and health behaviors and needs of FSWs in the two settings; qualitative interviews were conducted to understand rationales behind these behaviors and needs.

For peer review only - http://bmjopen.bmj.com/site/about/guidelines.xhtml

METHODS

Study setting

The ANRS 12361 PrEP-CI cross-sectional and mixed-methods study was designed and implemented with two Ivorian community-based organizations between September 2016 and March 2017. Aprosam works within the city of San Pedro and in the surrounding areas, particularly in villages close to farming businesses (coffee and cocoa exploitation). Espace Confiance operates in several districts of Abidjan, the economic capital of Côte d'Ivoire (Koumassi, Marcory, Treichville, Zone 4 and Port-Bouët with its beaches). Both of these nongovernmental organizations (NGOs) deliver HIV prevention and testing services directly at prostitution sites (outreach activities) and provide HIV and SRH care services through a community clinic, for MSM and FSWs. Recruitment of participants for this study was made possible by the Aprosam and Espace Confiance organizations' networks of peer educators and their access to the population. The purpose of the quantitative study was not to be representative of all FSWs in Côte d'Ivoire but rather to represent FSWs who could be reached by the two partner NGOs and who could potentially benefit from PrEP in a future program.

Quantitative analysis of a survey questionnaire

From October 2016 to January 2017, a convenience sample of 1000 FSWs were recruited either by peer educators or when FSWs visited NGOs' community clinics. Eligibility criteria for the quantitative survey included being 18 years or older, working at a prostitution site at the time of the survey, and never being diagnosed HIV-positive. Peer educators enrolled FSWs who met eligibility criteria and agreed to participate, after reading an information sheet and signing a consent form.

FSWs answered a face-to-face 45-item standardized paper questionnaire that assessed their sociodemographic characteristics (age, nationality, level of education, number of children), their sexual practices and behaviors (duration and location of sex work, usual price of sexual intercourses, condom use with clients and regular partners, assault/coerced sexual intercourse), their knowledge and use of community health services (medical consultations, hepatitis B immunization, declared STIs, sex work during menstruations, use and knowledge of contraception, undesired pregnancies, abortion), *a priori* acceptability of a PrEP offer (perception of the risk to contract HIV, knowledge of any medicine (traditional or modern) to prevent HIV infection, interest in a modern medicine for HIV prevention, acceptance of a medical follow-up every three months), HIV monitoring (regularity of HIV testing, knowledge of HIV status of regular partners). Data collection was carried out by peer educators in dedicated health centers and prostitutions sites.

FSWs were also tested for HIV and, in case of a positive result, dried blood spot (DBS) sampling was performed to determine the window of infection through a recent infection testing algorithm adapted to the Ivorian context and thus describe HIV incidence in this population.

We described the sociodemographic characteristics, sexual behaviors and reproductive health of participants surveyed according to the study setting (Abidjan and San Pedro). Due to the fact that it is not a randomly taken sample but rather a convenience sample of women reached by the two NGOs, statistical tests such as Pearson's chi-square test or Fisher's exact test could not be formally used to compare the two study settings. Missing data were excluded from percentage calculations. All analyses were performed with Stata software (StataTM 12.0 College Station, Texas, USA).

Qualitative analysis of interviews

In addition to the quantitative survey, a qualitative study was conducted from November 2016 to December 2016 among a convenience sample of 66 FSWs recruited during the outreach activities of peer educators and with the aim to reach a maximum of different profiles of women (in terms of age, number of years working as FSWs, type of prostitution site). On each prostitution site visited, we

For peer review only - http://bmjopen.bmj.com/site/about/guidelines.xhtml

decided to perform in-depth interviews or focus group discussions, depending on the practicality of the site (ambient noise, opportunity of privacy) and the time allowed. Every time, according to the type of interviews to be performed, one to three FSWS agreed to be interviewed individually or five to eight FSWs agreed to be interviewed as a group.

Data were collected at prostitution sites in and around Abidjan and San Pedro, by a female researcher in demography (first author) who carried out 22 in-depth interviews (duration: 30 to 60 minutes) and eight focus group discussions (FGDs) (duration: 60 to 150 minutes) using a semi-structured interview guide. FSWs were interviewed about their sociodemographic characteristics (age, nationality, level of education, number of children, number of dependents, partner/husband), sex work (entry into prostitution, duration and location of sex work, usual price of sexual intercourses, mobility, regular clients, work during menstruations, future perspectives), risky practices and sexual behaviors (condom use depending on the type of practices, main perceived risk of unprotected sex, current possession of condoms, negotiation of (un)protected sex with clients and regular partners), community dynamics (relationships with the pimp, the owner of the prostitution site, other FSWs, peer educators from NGOs, source of help in case of money, health or administrative issues), experiences with violence and discrimination (physical/moral violence from clients, partners, authorities, experiences of stigmatization, barriers in accessing health care or administrative procedures), knowledge and attitudes regarding HIV and STIs (perception of the risk to contract HIV depending on the type of practices, perception of the global risk to contract HIV and means used for prevention, frequency and location of HIV testing, physical signs of STIs and means used for treatment) , barriers to health care (untreated health issues, locations of care seeking), use of drugs and alcohol, barriers to and need for SRH services (knowledge and screening of cervical cancer, hepatitis B and C, tuberculosis, knowledge and use of contraception including emergency contraception, knowledge and recourse to social workers), *a priori* acceptability of a PrEP offer (knowledge of any medicine (traditional or modern) to prevent HIV infection, interest in a modern medicine for HIV prevention, acceptance of a medical follow-up every three months, issues arisen after the presentation of PrEP).

Each in-depth interview and FGD was recorded (except for two participants who refused it as they were afraid to be recognized), transcribed and uploaded into NVivo software (QSR International Pty Ltd. Version 11 Pro, 2016) by the qualitative interviewer. She also conducted the qualitative analysis following two principles. First, a cross-sectional review, based on questions derived from the discussion guide, allowed for a thematic analysis. Data collected provide great information on FSWs' sexual and health behaviors and needs, preferences and social trajectories. However, many themes are not included in this paper and will be addressed in a further paper. Main themes explored for this article were related to the access to care (visit of community health centers, use of mobile clinics, referral by peer educators, barriers in access to care and stigmatization) and to the potential interest, utility of PrEP and obstacles (unprotected sexual intercourses with clients and partners, risk perception and women's priorities, mobility and working periods, access to condoms, use of contraception, anticipation of high-risk sex and violent clients). We remained open to new themes as they emerged from the data in an inductive manner (e.g., condom breakage and self-medication). Second, we reviewed each interview or FGD as a whole in order to identify the chain of events leading each woman to not access healthcare or to not use condoms, for example. Quotes presented here were translated verbatim from French to English by the authors.

Patient and public involvement

No patient was involved in the research design nor in the conduct of the study. Peer educators of six different community NGOs were involved in the development of the research questions during a workshop. The two selected NGOs in Abidjan and San Pedro participated in the design, recruitment and conduct of the study. Data from the quantitative survey and qualitative interviews were

For peer review only - http://bmjopen.bmj.com/site/about/guidelines.xhtml

disseminated among the community through peer educators, who helped in the interpretation of results.

Ethical considerations

Research authorizations were obtained from the National Committee of Research Ethics within the Ivorian Ministry of Health and Public Hygiene (reference number: 057/MSHP/CNER-kp, delivered on June 28, 2016). Confidentiality was maintained, and data were anonymized. Informed consent was obtained by the investigator before each interview or questionnaire.

RESULTS

Main characteristics of participants

The characteristics of FSWs who participated in the quantitative survey are presented in Table 1. The median age was 25 (IQR=22-30) years in San Pedro and 24 (IQR=21-28) years in Abidjan. Compared to FSWs reached in Abidjan, those reached in San Pedro were less educated, more often Ivorian, more likely to be the mother of at least one child, were paid less money, and they worked less regularly but much more frequently in more than one city. FSWs in San Pedro were also more often in a relationship, and the interviews showed that their boyfriend was often their pimp.

Regarding the qualitative study, out of 66 interviewed FSWs, 26 agreed to provide their age: the median age was 28 (IQR=22-33) years. It became evident during the interview process that three FSWs were underage (<18). The interview participants were mostly Ivorian (n=44); the remaining third (n=22) was Nigerian.

Table 1. Main characteristics of participants in the quantitative survey

Variables	All women n (%) n=1000	San Pedro n (%) n=400	Abidjan n (%) n=600
**Median age (IQR) years**	25 [21 – 29]	25 [22 – 30]	24 [21 – 28]
**Age (years)**			
≤ 24	470 (47.0)	168 (42.0)	302 (50.3)
[25 – 34]	431 (43.1)	181 (45.2)	250 (41.7)
≥ 35	99 (9.9)	51 (12.8)	48 (8.0)
**Level of education**			
No school	220 (22.1)	115 (28.9)	105 (17.6)
Primary school	382 (38.4)	163 (40.9)	219 (36.7)
Secondary school/University	393 (39.5)	120 (30.2)	273 (45.7)
Missing	*5*	*2*	*3*
**Nationality**			
Ivorian	690 (69.0)	312 (78.0)	378 (63.0)
Foreign	310 (31.0)	88 (22.0)	222 (37.0)
**Has a boyfriend/husband**			
Yes	714 (71.9)	317 (80.7)	397 (66.2)
No	279 (28.1)	76 (19.3)	203 (33.8)
Missing	*7*	*7*	*0*
**Number of children**			
0	426 (43.1)	132 (33.3)	294 (49.7)
1	301 (30.5)	122 (30.8)	179 (30.2)
2	155 (15.7)	82 (20.7)	73 (12.3)
≥ 3	106 (10.7)	60 (15.2)	46 (7.8)
Missing	*12*	*4*	*8*
**Frequency of sex work**			
Every day or almost every day	743 (75.3)	275 (69.3)	468 (79.5)
Sometimes	243 (24.7)	122 (30.7)	121 (20.5)
Missing	*14*	*3*	*11*

**How many years sex work has been practiced**			
≤ 2	479 (47.9)	176 (44.0)	303 (50.5)
≥ 3	521 (52.1)	224 (56.0)	297 (49.5)
**Practiced sex work in more than one city**			
Yes	268 (26.9)	198 (49.7)	70 (11.7)
No	727 (73.1)	200 (50.3)	527 (88.3)
Missing	*5*	*2*	*3*
**Where/how clients are contacted[1]**			
Brothel	302 (30.2)	114 (28.5)	188 (31.3)
Beach	129 (12.9)	71 (17.7)	58 (9.7)
Bar/"maquis"	471 (47.1)	200 (50.0)	271 (45.2)
Street	145 (14.5)	47 (11.8)	98 (16.3)
By phone (through hotel owners)	216 (21.6)	123 (30.7)	93 (15.5)
Hotel	265 (26.5)	156 (39.0)	109 (18.2)
Home	131 (13.1)	66 (16.5)	65 (10.8)
**Number of clients during last day of work**			
≤ 4	706 (70.8)	233 (58.3)	473 (79.2)
≥ 5	291 (29.2)	167 (41.7)	124 (20.8)
Missing	*3*	*0*	*3*
**How much did the last client pay [in FCFA (USD)]**			
≤ 1999 (~3.50)	238 (23.8)	152 (38.0)	86 (14.3)
[2000 – 4999] (3.50 – 8.75)	287 (28.7)	138 (34.5)	149 (24.8)
[5000 – 9999] (8.75 – 17.50)	241 (24.1)	69 (17.2)	172 (28.7)
≥ 10000 (17.50)	234 (23.4)	41 (10.3)	193 (32.2)
**Ever suffered assault/coerced sexual intercourse**			
Yes	115 (11.7)	41 (10.5)	74 (12.6)
No	866 (88.3)	351 (89.5)	515 (87.4)
Missing	*19*	*8*	*11*

[1]Most FSWs meet clients in more than one location: the total is not equal to 100%.

High HIV exposure despite the use of condoms

Overall in the questionnaire, 79% of FSWs in San Pedro and 92% of FSWs in Abidjan reported consistent condom use with their clients (Table 2). However, the question about regular use of condoms could not fully capture actual condom use; there were several situations where FSWs had unprotected sexual intercourse. 23% would accept condomless sex for a large sum of money. This exposure to unprotected sex was reported as well by several FSWs during the qualitative interviews and was explained by the critical need for money.

"And when you look back at your week, you didn't even make 2000 francs. You begin to think about it. Ah! Honestly, I do accept [unprotected sex]." (FGD, San Pedro)

Several interviewed women also attested that violent clients had assaulted them and refused to use condoms.

"They brutalize us. Often, they don't wear any condom. They force us. Often even, young junkies, they can come upon us. And they assault us." (In-depth interview, San Pedro, 28 years old).

Moreover, 94% of FSWs in San Pedro and 89% of FSWs in Abidjan reported not systematically using condoms with their regular partner, even though only 10% and 21%, respectively, knew their partner's HIV status. This practice was reported during interviews as well, even though the women explained that they perceived a risk associated with condomless intercourse. During a focus group discussion that took place in a slum in San Pedro, above a bar where FSWs meet clients, interviewed women were discussing about their regular partners. One of them stated that her boyfriend asked her to not use any condoms to prove her trust.

"This guy, he tells you I'm faithful to you. I want us to have sex without condoms to show trust. That's why I think that the scary person is your boyfriend, not the client." (FGD, San Pedro)

For peer review only - http://bmjopen.bmj.com/site/about/guidelines.xhtml

Another one explained the lack of trust in her partners was balanced by the fact that they would protect her from violent clients.

"Love is the only weapon where you sleep with your enemy (laughs). I mean, he's your closest enemy. He's the one who can kill you because he's not with you only. But you say, he's my official. You need him because he protects you." (FGD, San Pedro)

So, if condom use was high in general, most FSWs were still exposed to HIV: 59% had at least one instance of condomless intercourse over the previous week. FSWs' responses to the first question assessing condom use might refer to "typical use" as opposed to specific circumstances.

Regarding HIV testing, 51% of FSWs in San Pedro and 43% of FSWS in Abidjan had received their last HIV test less than 6 months before. However, 7% in San Pedro and 15% in Abidjan had never been tested before the survey.

During each qualitative interview and FGD, we presented PrEP as a medicine that could protect them against HIV if properly taken and implying to have a regular medical follow-up. PrEP was not available in Côte d'Ivoire at the time of the interviews and women had never heard about it. However, several questions emerged in relation with concrete matters such as side-effects, cost, current availability in pharmacies, compatibility with pregnancy, appropriate reaction if one or more pills are forgotten, respect of the timing of daily administration, etc. Many interviewed women considered PrEP as useful to prevent HIV transmission from their regular partners in particular, as they felt obligated to not use any condoms with them.

"*Danger itself, it comes from the one beside me. That pill is welcome, because by taking it I protect myself against the one beside me*." (FGD, San Pedro)

PrEP was presented similarly, although more briefly, in the questionnaire. The large majority of surveyed FSWs (98.6%) showed interest in a medicine that could provide efficient protection against HIV. 99.4% of the FSWs interested in PrEP would agree to a medical follow-up every three months.

Table 2. Condom use and HIV exposure in the quantitative survey

Variables	All women n (%) n=1000	San Pedro n (%) n=400	Abidjan n (%) n=600
**Condom use with clients**			
Never	9 (0.9)	3 (0.8)	6 (1.0)
Sometimes	29 (3.0)	17 (4.4)	12 (2.1)
Often	86 (8.9)	58 (15.1)	28 (4.8)
Always	837 (87.0)	304 (79.2)	533 (92.1)
Does not know/does not want to answer	2 (0.1)	2 (0.5)	0 (0.0)
Missing	*37*	*16*	*21*
**Use of condom with boyfriend/husband**			
Never	370 (53.2)	157 (50.5)	213 (55.3)
Sometimes	115 (16.5)	61 (19.6)	54 (14.0)
Often	123 (17.7)	49 (15.8)	74 (19.2)
Always	62 (8.9)	19 (6.1)	43 (11.2)
Does not know/does not want to answer	26 (3.8)	25 (8.0)	1 (0.3)
Missing	*18*	*6*	*12*
**Acceptance of condomless sexual intercourse in exchange for a large sum of money**			
Never	764 (77.4)	251 (63.9)	513 (86.4)
Sometimes	79 (8.0)	52 (13.2)	27 (4.5)
Often	92 (9.3)	55 (14.0)	37 (6.2)
Always	12 (1.2)	6 (1.5)	6 (1.0)
Does not know/does not want to answer	40 (4.0)	29 (7.4)	11 (1.9)
Missing	*13*	*7*	*6*
**At least one instance of condomless intercourse over last 7 days**[1]			
Yes	220 (58.8)	152 (72.0)	68 (41.5)
No	154 (41.1)	59 (28.0)	95 (57.9)

For peer review only - http://bmjopen.bmj.com/site/about/guidelines.xhtml

Does not want to answer	1 (0.2)	0 (0.0)	1 (0.6)
Missing	*625*	*189*	*436*
**Last HIV test (months)**			
< 6	458 (45.9)	230 (50.9)	255 (42.6)
[6 – 12[	239 (24.0)	98 (24.6)	141 (23.6)
≥ 12	182 (18.2)	69 (17.3)	113 (18.9)
Never	114 (11.4)	26 (6.5)	88 (14.7)
Does not know/does not want to answer	4 (0.4)	3 (0.7)	1 (0.2)
Missing	*3*	*1*	*2*
**Knowledge of boyfriend/husband's HIV status**			
Yes	121 (17.4)	33 (10.6)	88 (23.0)
No	573 (82.3)	279 (89.1)	294 (768)
Does not want to answer	2 (0.3)	1 (0.3)	1 (0.3)
Missing	*18*	*4*	*14*
**Interest in a medicine protecting against HIV**			
Yes	982 (98.6)	394 (99.0)	588 (98.3)
No	11 (1.1)	2 (0.5)	9 (1.5)
Does not know	3 (0.3)	2 (0.5)	1 (0.2)
Missing	*4*	*2*	*2*
**If yes, would agree to a medical follow-up every 3 months**			
Yes	964 (99.4)	391 (99.7)	573 (99.1)
No	5 (0.5)	1 (0.3)	4 (0.7)
Does not know	1 (0.1)	0 (0.0)	1 (0.2)
Missing	*12*	*2*	*10*

[1]This variable was added during the survey; for this reason, some participants did not answer the question.

Beyond HIV, many unmet SRH needs exist

In total, 43% of the survey participants reported at least one unwanted pregnancy, and 50% had at least one abortion in their lifetime (Table 3). Only 39% of surveyed FSWs were using a contraceptive method other than condoms; among them, most FSWs in Abidjan mentioned taking the pill (70%) compared to only 33% of FSWs in San Pedro, where 35% declared using an implant. Unfortunately, child desire was not asked in the quantitative survey, which does not allow us to calculate the unmet need for contraception among FSWs. However, as a proxy, it appeared that most of the interviewed FSWs did not want a child at the moment.

"My main risk, it is to not get pregnant because I'm still a schoolgirl. If I get pregnant, who will take care of it [the baby]*?"* (In-depth interview, Abidjan, 18 years old)

However, women explained during interviews that they feared becoming sterile because of contraceptive means, especially the pill.

"They [peer educators from the community-based NGO] told me about the pill, but I refused because I don't have children yet. I don't want to have problems in the future." (In-depth interview, Abidjan, 18 years old)

"That's what makes me tired. I'm afraid because I don't have children yet. That's my problem, otherwise for diseases, well, there are condoms." (In-depth interview, San Pedro, 19 years old)

Moreover, only half of the survey participants knew about emergency contraception, among which 36% knew only nonmedical means.

Additionally, 36% of survey participants practiced sex work during menstruation, mainly using tampons (62%) or cold water (24%) to stop the bleeding.

Finally, 79% of FSWs in San Pedro and 55% of FSWs in Abidjan reported contracting an STI over the past 12 months. Even though half of the questionnaire survey participants thought they were very exposed to HIV infection, the interviewed FSWs often declared being preoccupied by other diseases as well, such as STIs or cancer.

For peer review only - http://bmjopen.bmj.com/site/about/guidelines.xhtml

"But we, every time, when we go in the bush, it's not only AIDS that kills. There are several diseases. Today we talk about cervical cancer. So I think it's not only AIDS we should get protected from. We have to protect ourselves from many diseases that are sexual." (FGD, San Pedro)

Despite the work of peer educators at prostitution sites, few surveyed FSWs visited the dedicated community clinics, with 76% in San Pedro and 61% in Abidjan consulting a health practitioner over the past year, among which 40% in San Pedro and 17% in Abidjan went to a dedicated facility. In interviews, some FSWs reported the inconvenient opening times and/or location, the fear of being identified as an FSW in the clinic area and the stigmatizing and judgmental attitudes of health professionals as reasons for not visiting these clinics. In the event of condom breakage, FSWs usually relied on self-medication. For example, a young woman described the beverages she would use for a vaginal douche.

"I'm going to buy Coke with Nescafe. It's for cleaning everything falling down." (In-depth interview, Abidjan, 17 years old)

They act similarly in case of suspicion of an STI.

"*Before going to the clinic, we try traditional plants and medicines first*." (FGD, San Pedro)

Table 3. Sexual and reproductive health in the quantitative survey

Variables	All women n (%) n=1000	San Pedro n (%) n=400	Abidjan n (%) n=600
**Had at least one undesired pregnancy**			
Yes	416 (42.9)	173 (45.1)	243 (41.5)
No	554 (57.1)	211 (54.9)	343 (58.5)
Missing	*30*	*16*	*14*
**Had at least one abortion**			
Yes	488 (50.2)	195 (50.4)	293 (50.0)
No	485 (49.8)	192 (49.6)	293 (50.0)
Missing	*27*	*13*	*14*
**Use of contraception other than condom**			
Yes	391 (39.1)	193 (48.3)	198 (33.0)
No	608 (60.8)	206 (51.5)	402 (67.0)
Does not know	1 (0.1)	1 (0.2)	0 (0.0)
**If yes, which contraceptive method[2]**			
Pill	204 (52.0)	65 (33.5)	139 (70.2)
Injectable	91 (23.3)	55 (28.5)	36 (18.2)
Implant	83 (21.2)	68 (35.1)	15 (7.6)
Other method[1] (nivaquine, spice, traditional medicine, etc.)	24 (6.1)	13 (6.7)	11 (5.6)
**Knowledge of emergency contraception**			
Yes	472 (48.4)	195 (50.8)	277 (46.9)
No	497 (51.0)	183 (47.7)	314 (53.1)
Does not know	6 (0.6)	6 (1.5)	0 (0.0)
Missing	*25*	*16*	*9*
**If yes, type of emergency contraception known[2]**			
Morning-after pill	304 (64.1)	100 (49.7)	204 (74.7)
Other[1] (antibiotic, coffee, soda, salted water, and lemon)	170 (35.9)	101 (50.3)	69 (25.3)
**Sex work during menstruation**			
Yes	363 (36.4)	154 (38.5)	209 (34.9)
No	635 (63.6)	246 (61.5)	389 (65.1)
Missing	*2*	*0*	*2*
**If yes, tool used for sex work during menstruation[2]**			
Wash with ice-cold water	86 (24.0)	37 (24.2)	49 (23.7)
Piece of ice	26 (7.3)	11 (7.2)	15 (7.3)
Tampon	222 (62.0)	105 (68.6)	117 (57.1)
Other tools[1] (hot water, soapy water, cotton, etc.)	49 (13.7)	12 (7.8)	37 (17.9)
**Self-reported STI (last 12 months)**			

For peer review only - http://bmjopen.bmj.com/site/about/guidelines.xhtml

Yes	639 (64.7)	312 (78.8)	327 (55.2)
No	349 (35.3)	84 (21.2)	265 (44.8)
Missing	*12*	*4*	*8*
**Last medical consultation**			
Less than 3 months	195 (19.6)	101 (25.4)	94 (15.7)
3 – 12 months	475 (47.7)	202 (50.8)	273 (45.6)
More than a year	258 (25.9)	79 (19.8)	179 (29.9)
Never consulted	68 (6.8)	16 (4.0)	52 (8.7)
Missing	*4*	*2*	*2*
**If ever consulted, site of the last consultation with a doctor/nurse**			
Dedicated facility	225 (26.3)	141 (40.5)	84 (16.6)
Public facility	415 (48.6)	123 (35.3)	292 (57.7)
Private facility	213 (24.9)	83 (23.8)	130 (25.7)
Does not know	1 (0.1)	1 (0.3)	0 (0.0)
Missing	*74*	*34*	*40*

[1]"Other" categories describe participants' specific answers.
[2]Several possible answers.

For peer review only

DISCUSSION

Both the quantitative and qualitative results showed that FSWs were highly exposed to HIV despite their use of condoms. There were in fact a variety of situations in which the surveyed FSWs had condomless sex. First, the large majority did not use condoms with their regular partner despite their acknowledged concurrent sexual partnerships. Some women experienced coercion on the part of their male partners, questioning their faith in the relationship; having condomless sex was a proof of trust that was difficult to negotiate. Others used condomless sex as a negotiation strategy to obtain protection from their partners against the threat of violence. In a context where gender norms reinforce male domination over women (20), they consciously took risks when facing the primacy of men's sexual pleasure (21). Second, some FSWs accepted condomless sexual intercourse for a large sum of money, especially when they had had few previous clients. Financial need associated with low prices of sexual intercourses and irregular weekly earnings drove some FSWs to engage in condomless sex as a way to earn more. Third, the violence or the threat thereof that FSWs faced sometimes prevented them to negotiate condom use. Different studies showed that women who are victims of abuse are less likely to use condoms with their clients than those who are not (22,23). Performing an illegal activity can also compel women, especially in the street, to negotiate quickly with clients at the expense of condom use. The situation appeared even worse in some rural areas around San Pedro, where the interviewed FSWs revealed that they could not buy any condoms in the village, as there was no point of sale.

Despite the adoption of preventive behavior (condom use) in most cases, FSWs are still highly exposed to HIV, due their high number of sexual partners and the occurrence of remaining unprotected sexual acts. In a complementary study within the PrEP-CI project (not yet published), we estimated the incidence among the 1000 surveyed FSWs using a recent infection testing algorithm adapted to the Ivorian context: we found an incidence of 2.2 per 100 person-years (1.5 in Abidjan and 3.2 in San Pedro) (24). In such context, oral PrEP could be an appropriate and complementary preventive tool to cover the situations where condom cannot be negotiated.

The majority of interviewed and surveyed FSWs had low awareness and knowledge of PrEP before our study but most of them were highly willing to use this medicine despite the constraint of regular medical follow-up. Participants felt PrEP would give added protection against infection, in particular with regular partners. A study in Kenya had similar findings and suggested to promote PrEP through outreach activities for sex workers (25). However, a PrEP implementation trial in South Africa showed

low adherence despite high declared acceptability before the implementation (18). For this reason we were attentive to challenges that might hinder PrEP uptake and adherence for FSWs, such as side-effects or timing of daily administration.

Our results showed that FSWs faced many unmet needs regarding SRH beyond HIV prevention and treatment. Inconsistent condom use exposed FSWs to STIs (26,27) and undesired pregnancies (28), which could increase their mortality and morbidity (29). The prevalence of contraceptive use was low in the surveyed population despite their high risk of undesired pregnancy due to the common fear of contraception causing sterility (30). Furthermore, using ice or tissues to continue sex work during menstruation has been proven to be a source of bacterial infections (31). These needs could be addressed in the community clinics of the two NGOs. However, as shown in other studies, FSWs faced many obstacles in accessing SRH care, because of the high costs or distance of the sites (32), the stigmatizing and discriminating attitudes of some health practitioners, the FSWs' social and economic marginalization, and restrictive laws related to their activity (33). FSWs thus preferred advice from their peers or self-medication. Moreover, peer educators from both NGOs expressed that public policies and international donors in Côte d'Ivoire currently focus on the identification and referral of new cases of HIV-positive FSWs, while HIV-negative women have limited access to care as stated above. A PrEP program requires a medical follow-up every three months and thus implies to consider the chronic follow-up of HIV-negative women. All efficacy PrEP trials provided a range of sexual healthcare services in addition to PrEP drugs. By design, these services were conditional to PrEP use. When transitioning to real life, such PrEP programs reproduced such service model. Our results suggest that a paradigm shift toward a patient-centered approach should be preferred, that is offering sexual and reproductive health services (such as contraception or STI testing and treatment) in which PrEP is an option but not mandatory. SRH services could also be a way to engage FSWs not ready for PrEP into regular care.

In addition, FSWs in San Pedro appeared to be in a more precarious situation than those in Abidjan due to their lower education level, higher number of children, irregularity of work, multiplicity of clients and work locations, and the lower price of sexual intercourse. They were also more likely to have condomless intercourse, notably for a large sum of money, and to report having had an STI over the past year. This can be explained by the fact that a large percentage of FSWs in San Pedro came to the area during the period of coffee and cocoa exploitation (September-December), which brought many migrant workers; this results in less stability and security. The high mobility of these women generates "seasons of risk" (34), i.e., times when an individual might face an increased risk of HIV infection. It is paramount to take this into account when implementing daily PrEP for these women (35); they are the population most likely to regularly suspend their PrEP use. Developing mobile clinics that deliver HIV and SRH care services directly at prostitution sites could mitigate the issue of FSWs' mobility and address barriers in access to care, such as distance between prostitution sites and clinics or stigmatization associated with their activity.

FSWs' needs for PrEP cannot be understood without additionally considering the broader contexts in which their risk of exposure to HIV is situated: the context of their work, their relationships and their concerns about family planning or stigmatization, etc. This is important information to consider if a PrEP program is to successfully serve this at-risk population. In order to address women's experiences and concerns, a global SRH care package delivered through both community clinics and mobile clinics on prostitution sites appear essential. Several studies related to PrEP and SRH needs of FSWs showed as well that combination prevention approaches are necessary. First, as pointed by a study in Zimbabwe (36), women need to perceive the risk of getting infected by HIV and to be able to access health services in order to take PrEP daily. Second, as shown by Dhana *et al.* in a systematic review

For peer review only - http://bmjopen.bmj.com/site/about/guidelines.xhtml

(37), there is a lack of coordination between SRH and HIV services dedicated to FSWs in Africa when they are two distinct services; HIV services delivery models should integrate SRH services. Furthermore, in order to minimize stigma related to entry into care, services for HIV-positive and services for HIV-negative should not be dissociated.

A strength of this study is the use of mixed-method allowing us to better describe and understand challenges of PrEP implementation among FSWs in Côte d'Ivoire, as well as the strong collaboration with two NGOs helping us to reach the FSWs. Yet, this study has some limitations. First, as it focused on FSWs reached by two NGOs, the included population was probably more likely to know about HIV prevention and to access SRH care. In addition, our sample did not include occasional or undeclared FSWs. As such, the results cannot be extrapolated to all FSWs working inside or around Abidjan and San Pedro but can provide an operational perspective for developing healthcare services. Peer-educators conducted the survey during their on-site activities, which already consistently extended their working time. For logistic reasons, it was not possible to monitor the number of FSWs present on site, potentially eligible, examined for eligibility and included in the survey. Therefore we are not able to provide participation rates. However, peer educators reported that most FSWs confirmed for eligibility did accept to answer the questionnaire. The survey was stopped when we reached the expected number of 1000 FSWs (600 in Abidjan and 400 in San Pedro) and all FSWs included in the survey were analyzed. Finally, the use of FGDs could have led to social desirable answers. A matter of concern, pointed out by field workers and data collected, are the young underage FSWs (three interviews were conducted with FSWs aged less than 18 on the beaches of Abidjan). Ivorian law authorizes HIV testing for teenagers aged 16 and 17, without parental consent. What about other care and services that cannot be delivered to them in the absence of consent?

CONCLUSIONS

Implementing PrEP among FSWs in West Africa, such as in Côte d'Ivoire, is not only about providing a new prevention tool but is also an invitation to consider the chronic follow-up of HIV-negative FSWs. A global care package should be offered to FSWs, including HIV prevention and care, STI screening and treatment, contraception, menstrual management counseling and HBV screening, vaccination and medical treatment. In addition, PrEP initiation should not limit access to SRH services; conversely, SRH services could be a way to attract FSWs to HIV prevention. Beyond reducing the risk of HIV among FSWs and their partners, PrEP provides an opportunity to improve their health condition more globally.

While current policies focus on only HIV-infected women and on the importance of testing new FSWs, our results highlight the importance of developing a people-focused approach, as opposed to an "HIV-focused approach", that integrates all SRH needs when transitioning from PrEP efficacy trials to implementation (19).

6. Conflict of interest statement

There are no conflicts of interest.

7. Contributorship Statement

JL, SE and CD designed the ANRS 12361 PrEP-CI study. JMM and MN implemented the quantitative survey with the support of CA and SK. VB conducted the qualitative interviews. VB and JL developed the research question addressed in this paper. VB did the qualitative analysis, and MN did the statistical analysis. VB wrote the manuscript with the support of JL, MP and MN. All authors contributed to the

For peer review only

interpretation and presentation of the findings. All authors approved the final version of the manuscript for submission.

8. Funding

The PrEP-CI ANRS 12361 was funded by the Bill and Melinda Gates Foundation (Investment ID: OPP1106343) and the French National Agency for AIDS and Viral Hepatitis Research (ANRS).

9. Acknowledgments

We would like to thank all participants as well as Aprosam's and Espace Confiance's peer educators.

10. Data sharing statement

Dataset are available upon request on Zenodo (DOI: 10.5281/zenodo.2269160).

11. References

1. Dwyer-Lindgren L, Cork MA, Sligar A, Steuben KM, Wilson KF, Provost NR, et al. Mapping HIV prevalence in sub-Saharan Africa between 2000 and 2017. Nature. 2019 Jun;570(7760):189–93.
2. Kharsany ABM, Karim QA. HIV Infection and AIDS in Sub-Saharan Africa: Current Status, Challenges and Opportunities. Open AIDS J. 2016 Apr 8;10:34–48.
3. Baggaley R, Hensen B, Ajose O, Grabbe K, Wong V, Schilsky A, et al. From caution to urgency: the evolution of HIV testing and counselling in Africa. Bulletin of the World Health Organization. 2012 Sep 1;90(9):652-658B.
4. World Health Organization. Consolidated Guidelines on Hiv Prevention, Diagnosis, Treatment and Care for Key Populations [Internet]. World Health Organization; 2016 [cited 2019 Sep 26]. Available from: http://proxy.library.carleton.ca/loginurl=https://www.deslibris.ca/ID/10063272
5. World Health Organization. Consolidated Guidelines on the Use of Antiretroviral Drugs for treating and preventing HIV infection. Recommendations for a Public Health Approach. Second Edition 2016. World Health Organization; 2016 p. 429.
6. UNAIDS. Factsheet - Global AIDS Update 2019. 2019 p. 6.
7. Molina J-M, Capitant C, Spire B, Pialoux G, Cotte L, Charreau I, et al. On-Demand Preexposure Prophylaxis in Men at High Risk for HIV-1 Infection. New England Journal of Medicine. 2015 Dec 3;373(23):2237–46.
8. Grant RM, Lama JR, Anderson PL, McMahan V, Liu AY, Vargas L, et al. Preexposure chemoprophylaxis for HIV prevention in men who have sex with men. N Engl J Med. 2010 Dec 30;363(27):2587–99.
9. Baeten JM, Donnell D, Ndase P, Mugo NR, Campbell JD, Wangisi J, et al. Antiretroviral Prophylaxis for HIV Prevention in Heterosexual Men and Women. New England Journal of Medicine. 2012 Aug 2;367(5):399–410.
10. WHO. Guideline on when to start antiretroviral therapy and on pre-exposure prophylaxis for HIV [Internet]. Geneva: World Health Organisation; 2015 Sep p. 78. Report No.: 978 92 4 150956 5. Available from: http://apps.who.int/iris/bitstream/10665/186275/1/9789241509565_eng.pdf
11. UNAIDS. UNAIDS data 2017 [Internet]. UNAIDS; 2017 [cited 2018 Jul 11]. Available from: http://www.unaids.org/sites/default/files/media_asset/2017_data-book_en.pdf
12. UNAIDS. On the Fast-Track to end AIDS by 2030: Focus on location and population [Internet]. 2015 p. 268. Available from: http://www.unaids.org/en/resources/documents/2015/FocusLocationPopulation
13. Corneli AL, Deese J, Wang M, Taylor D, Ahmed K, Agot K, et al. FEM-PrEP: Adherence Patterns and Factors Associated With Adherence to a Daily Oral Study Product for Pre-exposure Prophylaxis. J Acquir Immune Defic Syndr. 2014 Jul 1;66(3):324–31.

For peer review only - http://bmjopen.bmj.com/site/about/guidelines.xhtml

14. Marrazzo JM, Ramjee G, Richardson BA, Gomez K, Mgodi N, Nair G, et al. Tenofovir-based preexposure prophylaxis for HIV infection among African women. N Engl J Med. 2015 Feb 5;372(6):509–18.
15. Béhanzin L, Guédou FA, Geraldo N, Goma Matsétsé E, Aza-Gnandji M, Imorou Bah Chabi A, et al. PrEP and Early Antiretroviral Therapy Demonstration Project: Challenges to Ensure Follow-up and Adherence Among Female Sex Workers in Cotonou, Benin. In Chicago: HIVR4P 2016 Conference; 2016.
16. Alary M, Béhanzin L, Mboup A, Guédou FA, Geraldo N, Goma-Matsétsé E, et al. Early Antiretroviral Therapy and Daily Pre-exposure Prophylaxis for HIV Prevention Among Female Sex Workers in Cotonou, Benin: A Demonstration Study. In Madrid: HIVR4P 2016 Conference; 2018.
17. Eakle R, Gomez GB, Naicker N, Bothma R, Mbogua J, Cabrera Escobar MA, et al. HIV pre-exposure prophylaxis and early antiretroviral treatment among female sex workers in South Africa: Results from a prospective observational demonstration project. PLoS Med. 2017;14(11):1–17.
18. Eakle R, Bourne A, Mbogua J, Mutanha N, Rees H. Exploring acceptability of oral PrEP prior to implementation among female sex workers in South Africa. J Intern AIDS Soc. 2018 Feb 1;21(2):n/a-n/a.
19. Larmarange J, Becquet V, Masumbuko J-M, Nouaman M, Plazy M, Danel C, et al. Implementing preexposure prophylaxis among key populations: an opportunity for patient-centered services and management of hepatitis B. AIDS. 2018 Mar 27;32(6):829–30.
20. Deering KN, Shaw SY, Thompson LH, Ramanaik S, Raghavendra T, Doddamane M, et al. Fertility intentions, power relations and condom use within intimate and other non-paying partnerships of women in sex work in Bagalkot District, South India. AIDS Care. 2015 Oct 3;27(10):1241–9.
21. Ghimire L, Smith WCS, van Teijlingen ER, Dahal R, Luitel NP. Reasons for non- use of condoms and self- efficacy among female sex workers: a qualitative study in Nepal. BMC Women's Health [Internet]. 2011 Sep 26;11(42). Available from: https://doi.org/10.1186/1472-6874-11-42
22. Wirtz AL, Schwartz S, Ketende S, Anato S, Nadedjo FD, Ouedraogo HG, et al. Sexual Violence, Condom Negotiation, and Condom Use in the Context of Sex Work: Results From Two West African Countries. JAIDS Journal of Acquired Immune Deficiency Syndromes. 2015 Mar;68:S171.
23. Decker MR, McCauley HL, Phuengsamran D, Janyam S, Seage GR, Silverman JG. Violence victimisation, sexual risk and sexually transmitted infection symptoms among female sex workers in Thailand. Sexually Transmitted Infections. 2010 Jun 1;86(3):236–40.
24. Nouaman MN, Becquet V, Masumbuko J-M, Anoma C, Soh K, Plazy M, et al. Évaluation de l'incidence du VIH chez des travailleuses du sexe en Côte d'Ivoire (PrEP-CI ANRS 12361). In Bordeaux; 2018.
25. Restar AJ, Tocco JU, Mantell JE, Lafort Y, Gichangi P, Masvawure TB, et al. Perspectives on HIV Pre- and Post- Exposure Prophylaxes (PrEP and PEP) among Female and Male Sex Workers in Mombasa, Kenya: Implications for Integrating Biomedical Prevention into Sexual Health Services. AIDS Educ Prev. 2017 Apr;29(2):141–53.
26. Braunstein SL, Ingabire CM, Kestelyn E, Uwizera AU, Mwamarangwe L, Ntirushwa J, et al. High Human Immunodeficiency Virus Incidence in a Cohort of Rwandan Female Sex Workers. Sexually Transmitted Diseases. 2011 May;38(5):385.
27. Scorgie F, Chersich MF, Ntaganira I, Gerbase A, Lule F, Lo Y-R. Socio-Demographic Characteristics and Behavioral Risk Factors of Female Sex Workers in Sub-Saharan Africa: A Systematic Review. AIDS Behav. 2012 May 1;16(4):920–33.
28. Schwartz S, Papworth E, Thiam-Niangoin M, Abo K, Drame F, Diouf D, et al. An urgent need for integration of family planning services into HIV care: the high burden of unplanned pregnancy, termination of pregnancy, and limited contraception use among female sex workers in Côte d'Ivoire. JAIDS Journal of Acquired Immune Deficiency Syndromes. 2015 Mar 1;68(Supplement 3):S91–S98.
29. Mullick S. Sexually transmitted infections in pregnancy: prevalence, impact on pregnancy outcomes, and approach to treatment in developing countries. Sexually Transmitted Infections. 2005 Aug 1;81(4):294–302.

For peer review only - http://bmjopen.bmj.com/site/about/guidelines.xhtml

30. Guillaume A, Ltd. A. The Role of Abortion in the Fertility Transition in Abidjan (Côte d'Ivoire) during the 1990s. Population (English Edition, 2002-). 2003;58(6):657–85.
31. Baisley K, Changalucha J, Weiss HA, Mugeye K, Everett D, Hambleton I, et al. Bacterial vaginosis in female facility workers in north-western Tanzania: prevalence and risk factors. Sexually Transmitted Infections. 2009 Sep 1;85(5):370–5.
32. Wahed T, Alam A, Sultana S, Rahman M, Alam N, Martens M, et al. Barriers to sexual and reproductive healthcare services as experienced by female sex workers and service providers in Dhaka city, Bangladesh. PLoS ONE. 2017 31;12(7).
33. Ippoliti NB, Nanda G, Wilcher R. Meeting the Reproductive Health Needs of Female Key Populations Affected by HIV in Low-and Middle-Income Countries: A Review of the Evidence. Studies in Family Planning. 2017;48(2):121–151.
34. Elsesser SA, Oldenburg CE, Biello KB, Mimiaga MJ, Safren SA, Egan JE, et al. Seasons of Risk: Anticipated Behavior on Vacation and Interest in Episodic Antiretroviral Pre-exposure Prophylaxis (PrEP) Among a Large National Sample of U.S. Men Who have Sex with Men (MSM). AIDS Behav. 2016 Jul;20(7):1400–7.
35. Namey E, Agot K, Ahmed K, Odhiambo J, Skhosana J, Guest G, et al. When and why women might suspend PrEP use according to perceived seasons of risk: implications for PrEP-specific risk-reduction counselling. Culture, Health & Sexuality. 2016 Sep;18(9):1081–91.
36. Cowan FM, Delany-Moretlwe S. Promise and pitfalls of pre-exposure prophylaxis for female sex workers. Curr Opin HIV AIDS. 2016 Jan;11(1):27–34.
37. Dhana A, Luchters S, Moore L, Lafort Y, Roy A, Scorgie F, et al. Systematic review of facility-based sexual and reproductive health services for female sex workers in Africa. Globalization and Health. 2014 Jun 10;10:46.

For peer review only

For peer review only - http://bmjopen.bmj.com/site/about/guidelines.xhtml

STROBE Statement—Checklist of items that should be included in reports of ***cross-sectional studies***

	Item No	Recommendation	Reported on page #
**Title and abstract**	1	(*a*) Indicate the study's design with a commonly used term in the title or the abstract	1
		(*b*) Provide in the abstract an informative and balanced summary of what was done and what was found	2
**Introduction**			
Background/rationale	2	Explain the scientific background and rationale for the investigation being reported	3
Objectives	3	State specific objectives, including any prespecified hypotheses	3
**Methods**			
Study design	4	Present key elements of study design early in the paper	3
Setting	5	Describe the setting, locations, and relevant dates, including periods of recruitment, exposure, follow-up, and data collection	3-4
Participants	6	(*a*) Give the eligibility criteria, and the sources and methods of selection of participants	4
Variables	7	Clearly define all outcomes, exposures, predictors, potential confounders, and effect modifiers. Give diagnostic criteria, if applicable	4-5
Data sources/ measurement	8*	For each variable of interest, give sources of data and details of methods of assessment (measurement). Describe comparability of assessment methods if there is more than one group	5
Bias	9	Describe any efforts to address potential sources of bias	
Study size	10	Explain how the study size was arrived at	6
Quantitative variables	11	Explain how quantitative variables were handled in the analyses. If applicable, describe which groupings were chosen and why	5
Statistical methods	12	(*a*) Describe all statistical methods, including those used to control for confounding	5
		(*b*) Describe any methods used to examine subgroups and interactions	5
		(*c*) Explain how missing data were addressed	5
		(*d*) If applicable, describe analytical methods taking account of sampling strategy	
		(*e*) Describe any sensitivity analyses	
**Results**			
Participants	13*	(a) Report numbers of individuals at each stage of study—eg numbers potentially eligible, examined for eligibility, confirmed eligible, included in the study, completing follow-up, and analysed	6-7
		(b) Give reasons for non-participation at each stage	6-7
		(c) Consider use of a flow diagram	
Descriptive data	14*	(a) Give characteristics of study participants (eg demographic, clinical, social) and information on exposures and potential confounders	6-7
		(b) Indicate number of participants with missing data for each variable of interest	Cf. tables
Outcome data	15*	Report numbers of outcome events or summary measures	
Main results	16	(*a*) Give unadjusted estimates and, if applicable, confounder-adjusted estimates and their precision (eg, 95% confidence interval). Make clear which confounders were adjusted for and why they were included	Cf. tables

For peer review only - http://bmjopen.bmj.com/site/about/guidelines.xhtml

		(*b*) Report category boundaries when continuous variables were categorized	Cf. tables
		(*c*) If relevant, consider translating estimates of relative risk into absolute risk for a meaningful time period	
Other analyses	17	Report other analyses done—eg analyses of subgroups and interactions, and sensitivity analyses	
**Discussion**			
Key results	18	Summarise key results with reference to study objectives	10
Limitations	19	Discuss limitations of the study, taking into account sources of potential bias or imprecision. Discuss both direction and magnitude of any potential bias	11
Interpretation	20	Give a cautious overall interpretation of results considering objectives, limitations, multiplicity of analyses, results from similar studies, and other relevant evidence	10-11
Generalisability	21	Discuss the generalisability (external validity) of the study results	11
**Other information**			
Funding	22	Give the source of funding and the role of the funders for the present study and, if applicable, for the original study on which the present article is based	14

*Give information separately for exposed and unexposed groups.

Note: An Explanation and Elaboration article discusses each checklist item and gives methodological background and published examples of transparent reporting. The STROBE checklist is best used in conjunction with this article (freely available on the Web sites of PLoS Medicine at http://www.plosmedicine.org/, Annals of Internal Medicine at http://www.annals.org/, and Epidemiology at http://www.epidem.com/). Information on the STROBE Initiative is available at www.strobe-statement.org.

For peer review only

For peer review only - http://bmjopen.bmj.com/site/about/guidelines.xhtml

BMJ Open

Sexual health needs of female sex workers in Côte d'Ivoire: a mixed-methods study to prepare the future implementation of pre-exposure prophylaxis (PrEP) for HIV prevention

Journal:	*BMJ Open*
Manuscript ID	bmjopen-2018-028508.R2
Article Type:	Original research
Date Submitted by the Author:	05-Dec-2019

Complete List of Authors:	Becquet, Valentine; INED; Centre Population et Développement, Institut de Recherche pour le Développement, Université Paris Descartes, ERL Inserm U 1244<br>Nouaman, Marcellin; Programme PAC-CI/ANRS Research Site<br>Plazy, Mélanie; Inserm Research Center 1219 (Bordeaux Population Health), ISPED, Université de Bordeaux<br>Masumbuko, Jean-Marie; Programme PAC-CI/ANRS Research Site<br>Anoma, Camille; Espace Confiance<br>Kouame, Soh; Aprosam<br>Danel, Christine; Programme PAC-CI/ANRS Research Site<br>Eholie, Serge; Programme PAC-CI/ANRS Research Site<br>Larmarange, Joseph; Centre Population et Développement, Institut de Recherche pour le Développement, Université Paris Descartes, ERL Inserm U 1244

**Primary Subject Heading**:	Sexual health
Secondary Subject Heading:	HIV/AIDS, Qualitative research
Keywords:	QUALITATIVE RESEARCH, HIV & AIDS < INFECTIOUS DISEASES, Public health < INFECTIOUS DISEASES, Demography < TROPICAL MEDICINE, Epidemiology < TROPICAL MEDICINE

SCHOLARONE™ Manuscripts

1. Title page

Sexual health needs of female sex workers in Côte d'Ivoire: a mixed-methods study to prepare the future implementation of pre-exposure prophylaxis (PrEP) for HIV prevention

Valentine Becquet[1,2§], Marcellin Nouaman[3], Mélanie Plazy[4], Jean-Marie Masumbuko[3], Camille Anoma[5], Soh Kouamé[6], Christine Danel[3], Serge Eholié[3], Joseph Larmarange[2] for the ANRS 12361 PrEP-CI Study group
[1]French Institute for Demographic Studies (INED), Paris, France
[2]Centre Population et Développement (CEPED), French Institute for Research on Sustainable Development (IRD), Université de Paris, ERL Inserm U 1244, Paris, France
[3]Programme PAC-CI site ANRS de Côte d'Ivoire, Abidjan, Côte d'Ivoire
[4]Inserm Research Center 1219 (Bordeaux Population Health), ISPED, Université de Bordeaux, Bordeaux, France
[5]Espace Confiance, Abidjan, Côte d'Ivoire
[6]Aprosam, San Pedro, Côte d'Ivoire
§ Correspondance to Dr. Valentine Becquet, valentine.becquet@ined.fr
Email addresses:
VB: valentine.becquet@ined.fr
MN: nouaman_et_vie@yahoo.fr
MP: melanie.plazy@u-bordeaux.fr
JMM: masumbukojm@gmail.com
CA: anomacamille@yahoo.fr
SK: sohkouame1@yahoo.fr
CD: christinemarie.danel@gmail.com
SE: sergeholie@yahoo.fr
JL: joseph.larmarange@ceped.org

Members of the ANRS 12361 PrEP-CI study group:

- ABOUBAKAR Sangaré (Aprosam, San Pedro, Côte d'Ivoire)
- ANGLARET Xavier (PAC-CI, Abidjan, Côte d'Ivoire / Inserm, Bordeaux, France)
- ANOMA Camille (Espace Confiance, Abidjan, Côte d'Ivoire)
- BARIN Francis (Université François Rabelais, Tours, France)
- BAZIN Brigitte (ANRS, Paris, France)
- BECQUET Valentine (Ceped/IRD, Paris, France)
- DABIS François (ISPED/Inserm, Bordeaux, France)
- DANEL Christine (PAC-CI, Abidjan, Côte d'Ivoire / Inserm, Bordeaux, France)
- EHOLIE Serge (PAC-CI, Abidjan, Côte d'Ivoire)
- EKOUEVI Didier (PAC-CI, Abidjan, Côte d'Ivoire)
- FONSART Julien (Hôpital Saint-Louis, Paris, France)
- GBOSI Kate (Aprosam, San Pedro, Côte d'Ivoire)
- KWAMÉ Abo (Programme National de Lutte contre le Sida, Côte d'Ivoire)
- LARMARANGE Joseph (Ceped/IRD, Paris, France)
- MASUMBUKO Jean-Marie (PAC-CI, Abidjan, Côte d'Ivoire)

For peer review only - http://bmjopen.bmj.com/site/about/guidelines.xhtml

- MÉDA Nicolas (Centre Muraz, Bobo-Dioulasso, Burkina Faso)
- MOH Raoul (PAC-CI, Abidjan, Côte d'Ivoire)
- MOLINA Jean-Michel (Hôpital Saint-Louis, Paris, France)
- N'DRI-YOMAN Thérèse (PAC-CI, Abidjan, Côte d'Ivoire)
- NOUAMAN Marcellin (PAC-CI, Abidjan, Côte d'Ivoire)
- PLAZY Mélanie (ISPED / Inserm, Bordeaux, France)
- SOH Kouamé (Aprosam, San Pedro, Côte d'Ivoire)
- TANOE Solange (Espace Confiance, Abidjan, Côte d'Ivoire)
- YEO Roselyne (Espace Confiance, Abidjan, Côte d'Ivoire)

Word count : 4957

For peer review only

For peer review only - http://bmjopen.bmj.com/site/about/guidelines.xhtml

2. Keywords (6)

HIV prevention; sexual and reproductive health; people-focused approach; pre-exposure prophylaxis (PrEP); sex work; mixed-methods research

3. Abstract

Objective

Describe sexual and reproductive health (SRH) needs of female sex workers (FSWs) to inform the future implementation of pre-exposure prophylaxis (PrEP) for HIV prevention in this population.

Design and setting

The ANRS 12361 PrEP-CI cross-sectional and mixed-methods study was designed and implemented with two community-based organizations in Côte d'Ivoire.

Participants

A convenience sample of 1000 FSWs aged ≥18, not known as HIV+, completed a standardized questionnaire assessing sociodemographic characteristics, sexual practices, use of community health services, and *a priori* acceptability of PrEP.

Twenty-two in-depth interviews and eight focus-group discussions were also conducted to document FSWs' risky practices and sexual behaviors, experiences with violence and discrimination, attitudes regarding HIV and sexually transmitted infections (STIs), and barriers to SRH services.

Results

Although 87% described consistent condom use with clients, >22% declared accepting condomless sexual intercourse for a large sum of money. Furthermore, condom use with their steady partner and knowledge of their partner's HIV status was low, despite their acknowledged concurrent sexual partnerships.

While inconsistent condom use exposed FSWs to STIs and undesired pregnancies, the prevalence of contraceptive strategies other than condoms was low (39%) due to fear of contraception causing sterility. FSWs faced obstacles in accessing SRH care and preferred advice from their peers or self-medication.

Conclusions

Despite adoption of preventive behavior in most cases, FSWs are still highly exposed to HIV. Furthermore, FSWs seem to face several barriers in accessing SRH. Implementing PrEP among FSWs in West Africa, such as in Côte d'Ivoire, constitutes an opportunity to consider the chronic follow-up of HIV-negative FSWs. PrEP initiation should not condition access to SRH services; conversely, SRH services could be a way to attract FSWs into HIV prevention. Our results highlight the importance of developing a people-focused approach that integrates all SRH needs when transitioning from PrEP efficacy trials to implementation.

4. Article Summary

Strengths and limitations of this study

- Combination of a quantitative survey to document sexual and health behaviors and needs of FSWs and of a qualitative survey to understand rationales behind these behaviors and needs
- Use of a convenience sample representative of FSWs actually reached by two community NGOs but not representative of the overall population of FSWs
- A comprehensive descriptive study but not powered enough for multivariate explicative analysis

For peer review only - http://bmjopen.bmj.com/site/about/guidelines.xhtml

5. Main text

INTRODUCTION

Despite global progress in reducing new HIV infections and AIDS-related deaths in the last 10 years in sub-Saharan Africa (1,2), current policies and programmes are focusing on the identification of HIV-infected people in order to link them to HIV care and treatment (3,4), knowing that antiretroviral treatment has been proven to reduce HIV transmission (5). However, the number of new HIV infections still remain too high to achieve epidemic control (6). Numerous trials have shown that oral preexposure prophylaxis (PrEP), when taken correctly, was highly efficacious to prevent HIV acquisition, in particular among men having sex with men (MSM) (7–9). Since 2015, oral PrEP has been recommended by the World Health Organisation (WHO) for populations at "substantial risk" of HIV acquisition (10).

In West Africa, most countries have mixed HIV epidemics, with a relatively low prevalence in the general population (compared to that in Eastern and Southern Africa) but severely affected key populations, particularly female sex workers (FSWs) and MSM (11). In Côte d'Ivoire in 2012, the HIV prevalence was estimated to be 29% in FSWs (12) and 19% among MSM (12). The National Program against HIV/AIDS (PNLS) requested operational research and data on the relevance of PrEP in order to better consider its future integration in the national algorithm of HIV prevention. A PrEP demonstration project (ANRS 12324 CohMSM) is currently being implemented among MSM. Our research team was invited to explore the sexual health needs of FSWs in this country as a potential target for a future PrEP program, knowing that PrEP was not yet available.

Although PrEP is effective when taken properly, the FEM-PREP (13) and VOICE (14) trials conducted among women from the general population in Southern and Eastern Africa showed low adherence to the treatment, resulting in a low or even null effect of PrEP. Similarly, PrEP implementation trials conducted among FSWs in Africa showed varying results regarding retention. In Benin, the retention rate after 10 months was 66% (15) and the overall retention rate after a complete follow-up of 28 months was 48% (16). In South Africa, it was 22% after 12 months (17) despite a high declared acceptability of PrEP before the implementation (18). Moreover, PrEP constitutes a new HIV prevention tool but does not prevent sexually transmitted infections (STIs) or unwanted pregnancies. It is therefore necessary to consider the overall needs of target populations in terms of sexual and reproductive health (SRH) (19). More operational and social science research is needed for the implementation of PrEP to be a success, especially on creating demand for oral PrEP; improving adherence; understanding the social and behavioral impact of PrEP; and integrating PrEP services with other services (10).

In this context, in order to design a future PrEP program targeting FSWs, the ANRS 12361 PrEP-CI pilot study was implemented to explore sexual health care needs that should be considered within such a program and to better describe FSWs currently reached by peer educators. This paper aims to describe the work and social environment of FSWs, their SRH needs and possible barriers for accessing care in two different settings in Côte d'Ivoire; i.e. different elements that need to be taken into account when implementing PrEP. We adopted a mixed approach: a quantitative survey was used to reach a high number of FSWs in order to be able to calculate incidence of HIV infection and to compare sexual and health behaviors and needs of FSWs in the two settings; qualitative interviews were conducted to understand rationales behind these behaviors and needs.

For peer review only - http://bmjopen.bmj.com/site/about/guidelines.xhtml

METHODS

Study setting

The ANRS 12361 PrEP-CI cross-sectional and mixed-methods study was designed and implemented with two Ivorian community-based organizations between September 2016 and March 2017. Aprosam works within the city of San Pedro and in the surrounding areas, particularly in villages close to farming businesses (coffee and cocoa exploitation). Espace Confiance operates in several districts of Abidjan, the economic capital of Côte d'Ivoire (Koumassi, Marcory, Treichville, Zone 4 and Port-Bouët with its beaches). Both of these nongovernmental organizations (NGOs) deliver HIV prevention and testing services directly at prostitution sites (outreach activities) and provide HIV and SRH care services through a community clinic, for MSM and FSWs. Recruitment of participants for this study was made possible by the Aprosam and Espace Confiance organizations' networks of peer educators and their access to the population. The purpose of the quantitative study was not to be representative of all FSWs in Côte d'Ivoire but rather to represent FSWs who could be reached by the two partner NGOs and who could potentially benefit from PrEP in a future program.

Quantitative analysis of a survey questionnaire

From October 2016 to January 2017, a convenience sample of 1000 FSWs were recruited either by peer educators or when FSWs visited NGOs' community clinics. Eligibility criteria for the quantitative survey included being 18 years or older, working at a prostitution site at the time of the survey, and being HIV negative or of unknown HIV status at the time of the survey. Peer educators enrolled FSWs who met eligibility criteria and agreed to participate, after reading an information sheet and signing a consent form.

FSWs answered a face-to-face 45-item standardized paper questionnaire that assessed their sociodemographic characteristics (age, nationality, level of education, number of children), their sexual practices and behaviors (duration and location of sex work, usual price of sexual intercourses, condom use with clients and regular partners, assault/coerced sexual intercourse), their knowledge and use of community health services (medical consultations, hepatitis B immunization, declared STIs, sex work during menstruations, use and knowledge of contraception, undesired pregnancies, abortion), *a priori* acceptability of a PrEP offer (perception of the risk to contract HIV, knowledge of any medicine (traditional or modern) to prevent HIV infection, interest in a modern medicine for HIV prevention, acceptance of a medical follow-up every three months), HIV monitoring (regularity of HIV testing, knowledge of HIV status of regular partners). Data collection was carried out by peer educators in dedicated health centers and prostitutions sites.

FSWs were also tested for HIV and, in case of a positive result, dried blood spot (DBS) sampling was performed to determine the window of infection through a recent infection testing algorithm adapted to the Ivorian context and thus describe HIV incidence in this population. Individuals diagnosed with HIV during the study were referred to the community clinics by peer educators, for HIV care and treatment.

We described the sociodemographic characteristics, sexual behaviors and reproductive health of participants surveyed according to the study setting (Abidjan and San Pedro). Due to the fact that it is not a randomly taken sample but rather a convenience sample of women reached by the two NGOs, statistical tests such as Pearson's chi-square test or Fisher's exact test could not be formally used to compare the two study settings. Missing data were excluded from percentage calculations. All analyses were performed with Stata software (StataTM 12.0 College Station, Texas, USA).

For peer review only - http://bmjopen.bmj.com/site/about/guidelines.xhtml

Qualitative analysis of interviews

In addition to the quantitative survey, a qualitative study was conducted from November 2016 to December 2016 among a convenience sample of 66 FSWs recruited during the outreach activities of peer educators and with the aim to reach a maximum of different profiles of women (in terms of age, number of years working as FSWs, type of prostitution site). On each prostitution site visited, we decided to perform in-depth interviews or focus group discussions, depending on the practicality of the site (ambient noise, opportunity of privacy) and the time allowed. Every time, according to the type of interviews to be performed, we conducted one to three individual interviews and/or one focus group with five to eight FSWs.

Data were collected at prostitution sites in and around Abidjan and San Pedro, by a female researcher in demography (first author) who carried out 22 in-depth interviews (duration: 30 to 60 minutes) and eight focus group discussions (FGDs) (duration: 60 to 150 minutes) using a semi-structured interview guide. FSWs were interviewed about their sociodemographic characteristics (age, nationality, level of education, number of children, number of dependents, partner/husband), sex work (entry into prostitution, duration and location of sex work, usual price of sexual intercourses, mobility, regular clients, work during menstruations, future perspectives), risky practices and sexual behaviors (condom use depending on the type of practices, main perceived risk of unprotected sex, current possession of condoms, negotiation of (un)protected sex with clients and regular partners), community dynamics (relationships with the pimp, the owner of the prostitution site, other FSWs, peer educators from NGOs, source of help in case of money, health or administrative issues), experiences with violence and discrimination (physical/moral violence from clients, partners, authorities, experiences of stigmatization, barriers in accessing health care or administrative procedures), knowledge and attitudes regarding HIV and STIs (perception of the risk to contract HIV depending on the type of practices, perception of the global risk to contract HIV and means used for prevention, frequency and location of HIV testing, physical signs of STIs and means used for treatment) , barriers to health care (untreated health issues, locations of care seeking), use of drugs and alcohol, barriers to and need for SRH services (knowledge and screening of cervical cancer, hepatitis B and C, tuberculosis, knowledge and use of contraception including emergency contraception, knowledge and recourse to social workers), *a priori* acceptability of a PrEP offer (knowledge of any medicine (traditional or modern) to prevent HIV infection, interest in a modern medicine for HIV prevention, acceptance of a medical follow-up every three months, issues arisen after the presentation of PrEP).

Each in-depth interview and FGD was recorded (except for two participants who refused it as they were afraid to be recognized), transcribed and uploaded into NVivo software (QSR International Pty Ltd. Version 11 Pro, 2016) by the qualitative interviewer. She also conducted the qualitative analysis following two principles. First, a cross-sectional review, based on questions derived from the discussion guide, allowed for a thematic analysis. Data collected provide great information on FSWs' sexual and health behaviors and needs, preferences and social trajectories. However, many themes are not included in this paper and will be addressed in a further paper. Main themes explored for this article were related to the access to care (visit of community health centers, use of mobile clinics, referral by peer educators, barriers in access to care and stigmatization) and to the potential interest, utility of PrEP and obstacles (unprotected sexual intercourses with clients and partners, risk perception and women's priorities, mobility and working periods, access to condoms, use of contraception, anticipation of high-risk sex and violent clients). We remained open to new themes as they emerged from the data in an inductive manner (e.g., condom breakage and self-medication). Second, we reviewed each interview or FGD as a whole in order to identify the chain of events leading each woman to not access healthcare or to not use condoms, for example. Quotes presented here were translated verbatim from French to English by the authors.

For peer review only - http://bmjopen.bmj.com/site/about/guidelines.xhtml

Patient and public involvement

No patient was involved in the research design nor in the conduct of the study. Peer educators of six different community NGOs were involved in the development of the research questions during a workshop. The two selected NGOs in Abidjan and San Pedro participated in the design, recruitment and conduct of the study. Data from the quantitative survey and qualitative interviews were disseminated among the community through peer educators, who helped in the interpretation of results.

Ethical considerations

Research authorizations were obtained from the National Committee of Research Ethics within the Ivorian Ministry of Health and Public Hygiene (reference number: 057/MSHP/CNER-kp, delivered on June 28, 2016). Confidentiality was maintained, and data were anonymized. Written informed consent was obtained by the investigator before each interview or questionnaire.

RESULTS

Main characteristics of participants

The characteristics of FSWs who participated in the quantitative survey are presented in Table 1. The median age was 25 (IQR=22-30) years in San Pedro and 24 (IQR=21-28) years in Abidjan. Compared to FSWs reached in Abidjan, those reached in San Pedro were less educated, more often Ivorian, more likely to be the mother of at least one child, were paid less money, and they worked less regularly but much more frequently in more than one city. FSWs in San Pedro were also more often in a relationship, and the interviews showed that their boyfriend was often their pimp.
Regarding the qualitative study, out of 66 interviewed FSWs, 26 agreed to provide their age: the median age was 28 (IQR=22-33) years. It became evident during the interview process that three FSWs were underage (<18). The interview participants were mostly Ivorian (n=44); the remaining third (n=22) was Nigerian.

Table 1. Main characteristics of participants in the quantitative survey

Variables	All women n (%) n=1000	San Pedro n (%) n=400	Abidjan n (%) n=600
**Median age (IQR) years**	25 [21 – 29]	25 [22 – 30]	24 [21 – 28]
**Age (years)**			
≤ 24	470 (47.0)	168 (42.0)	302 (50.3)
[25 – 34]	431 (43.1)	181 (45.2)	250 (41.7)
≥ 35	99 (9.9)	51 (12.8)	48 (8.0)
**Level of education**			
No school	220 (22.1)	115 (28.9)	105 (17.6)
Primary school	382 (38.4)	163 (40.9)	219 (36.7)
Secondary school/University	393 (39.5)	120 (30.2)	273 (45.7)
Missing	*5*	*2*	*3*
**Nationality**			
Ivorian	690 (69.0)	312 (78.0)	378 (63.0)
Foreign	310 (31.0)	88 (22.0)	222 (37.0)
**Has a boyfriend/husband**			
Yes	714 (71.9)	317 (80.7)	397 (66.2)
No	279 (28.1)	76 (19.3)	203 (33.8)
Missing	*7*	*7*	*0*
**Number of children**			
0	426 (43.1)	132 (33.3)	294 (49.7)
1	301 (30.5)	122 (30.8)	179 (30.2)
2	155 (15.7)	82 (20.7)	73 (12.3)

For peer review only - http://bmjopen.bmj.com/site/about/guidelines.xhtml

≥ 3	106 (10.7)	60 (15.2)	46 (7.8)
Missing	*12*	*4*	*8*
**Frequency of sex work**			
Every day or almost every day	743 (75.3)	275 (69.3)	468 (79.5)
Sometimes	243 (24.7)	122 (30.7)	121 (20.5)
Missing	*14*	*3*	*11*
**How many years sex work has been practiced**			
≤ 2	479 (47.9)	176 (44.0)	303 (50.5)
≥ 3	521 (52.1)	224 (56.0)	297 (49.5)
**Practiced sex work in more than one city**			
Yes	268 (26.9)	198 (49.7)	70 (11.7)
No	727 (73.1)	200 (50.3)	527 (88.3)
Missing	*5*	*2*	*3*
**Where/how clients are contacted[1]**			
Brothel	302 (30.2)	114 (28.5)	188 (31.3)
Beach	129 (12.9)	71 (17.7)	58 (9.7)
Bar/“maquis”	471 (47.1)	200 (50.0)	271 (45.2)
Street	145 (14.5)	47 (11.8)	98 (16.3)
By phone (through hotel owners)	216 (21.6)	123 (30.7)	93 (15.5)
Hotel	265 (26.5)	156 (39.0)	109 (18.2)
Home	131 (13.1)	66 (16.5)	65 (10.8)
**Number of clients during last day of work**			
≤ 4	706 (70.8)	233 (58.3)	473 (79.2)
≥ 5	291 (29.2)	167 (41.7)	124 (20.8)
Missing	*3*	*0*	*3*
**How much did the last client pay [in FCFA (USD)]**			
≤ 1999 (~3.50)	238 (23.8)	152 (38.0)	86 (14.3)
[2000 – 4999] (3.50 – 8.75)	287 (28.7)	138 (34.5)	149 (24.8)
[5000 – 9999] (8.75 – 17.50)	241 (24.1)	69 (17.2)	172 (28.7)
≥ 10000 (17.50)	234 (23.4)	41 (10.3)	193 (32.2)
**Ever suffered assault/coerced sexual intercourse**			
Yes	115 (11.7)	41 (10.5)	74 (12.6)
No	866 (88.3)	351 (89.5)	515 (87.4)
Missing	*19*	*8*	*11*

[1]Most FSWs meet clients in more than one location: the total is not equal to 100%.

High HIV exposure despite the use of condoms

Overall in the questionnaire, 79% of FSWs in San Pedro and 92% of FSWs in Abidjan reported consistent condom use with their clients (Table 2). However, the question about regular use of condoms could not fully capture actual condom use; there were several situations where FSWs had unprotected sexual intercourse. 23% would accept condomless sex for a large sum of money. This exposure to unprotected sex was reported as well by several FSWs during the qualitative interviews and was explained by the critical need for money.

“And when you look back at your week, you didn’t even make 2000 francs. You begin to think about it. Ah! Honestly, I do accept [unprotected sex].” (FGD, San Pedro)

Several interviewed women also attested that violent clients had assaulted them and refused to use condoms.

“They brutalize us. Often, they don’t wear any condom. They force us. Often even, young junkies, they can come upon us. And they assault us.” (In-depth interview, San Pedro, 28 years old).

Moreover, 94% of FSWs in San Pedro and 89% of FSWs in Abidjan reported not systematically using condoms with their regular partner, even though only 10% and 21%, respectively, knew their partner’s HIV status. This practice was reported during interviews as well, even though the women explained that they perceived a risk associated with condomless intercourse. During a focus group discussion

For peer review only - http://bmjopen.bmj.com/site/about/guidelines.xhtml

that took place in a slum in San Pedro, above a bar where FSWs meet clients, interviewed women were discussing about their regular partners. One of them stated that her boyfriend asked her to not use any condoms to prove her trust.

"This guy, he tells you I'm faithful to you. I want us to have sex without condoms to show trust. That's why I think that the scary person is your boyfriend, not the client." (FGD, San Pedro)

Another one explained the lack of trust in her partners was balanced by the fact that they would protect her from violent clients.

"Love is the only weapon where you sleep with your enemy (laughs). I mean, he's your closest enemy. He's the one who can kill you because he's not with you only. But you say, he's my official. You need him because he protects you." (FGD, San Pedro)

Even if the majority of FSWs declared regular condom use, most of them were still exposed to HIV: 59% had at least one instance of condomless intercourse over the previous week. FSWs' responses to the first question assessing condom use might refer to "typical use" as opposed to specific circumstances.

Regarding HIV testing, 51% of FSWs in San Pedro and 43% of FSWS in Abidjan had received their last HIV test less than 6 months before. However, 7% in San Pedro and 15% in Abidjan had never been tested before the survey.

During each qualitative interview and FGD, we presented PrEP as a medicine that could protect against HIV if properly taken, and explained that it would require regular medical follow-up. PrEP was not available in Côte d'Ivoire at the time of the interviews and women had never heard about it. However, several questions emerged in relation with concrete matters such as side-effects, cost, current availability in pharmacies, compatibility with pregnancy, appropriate reaction if one or more pills are forgotten, respect of the timing of daily administration, etc. Many interviewed women considered PrEP as useful to prevent HIV transmission from their regular partners in particular, as they felt obligated to not use any condoms with them.

"Danger itself, it comes from the one beside me. That pill is welcome, because by taking it I protect myself against the one beside me." (FGD, San Pedro)

PrEP was presented similarly, although more briefly, in the questionnaire. The large majority of surveyed FSWs (98.6%) showed interest in a medicine that could provide efficient protection against HIV. 99.4% of the FSWs interested in PrEP would agree to a medical follow-up every three months.

Table 2. Condom use and HIV exposure in the quantitative survey

Variables	All women n (%) n=1000	San Pedro n (%) n=400	Abidjan n (%) n=600
**Condom use with clients**			
Never	9 (0.9)	3 (0.8)	6 (1.0)
Sometimes	29 (3.0)	17 (4.4)	12 (2.1)
Often	86 (8.9)	58 (15.1)	28 (4.8)
Always	837 (87.0)	304 (79.2)	533 (92.1)
Does not know/does not want to answer	2 (0.1)	2 (0.5)	0 (0.0)
Missing	*37*	*16*	*21*
**Use of condom with boyfriend/husband**			
Never	370 (53.2)	157 (50.5)	213 (55.3)
Sometimes	115 (16.5)	61 (19.6)	54 (14.0)
Often	123 (17.7)	49 (15.8)	74 (19.2)
Always	62 (8.9)	19 (6.1)	43 (11.2)
Does not know/does not want to answer	26 (3.8)	25 (8.0)	1 (0.3)
Missing	*18*	*6*	*12*
**Acceptance of condomless sexual intercourse in exchange for a large sum of money**			
Never	764 (77.4)	251 (63.9)	513 (86.4)
Sometimes	79 (8.0)	52 (13.2)	27 (4.5)

For peer review only - http://bmjopen.bmj.com/site/about/guidelines.xhtml

Often	92 (9.3)	55 (14.0)	37 (6.2)
Always	12 (1.2)	6 (1.5)	6 (1.0)
Does not know/does not want to answer	40 (4.0)	29 (7.4)	11 (1.9)
Missing	*13*	*7*	*6*
**At least one instance of condomless intercourse over last 7 days**[1]			
Yes	220 (58.8)	152 (72.0)	68 (41.5)
No	154 (41.1)	59 (28.0)	95 (57.9)
Does not want to answer	1 (0.2)	0 (0.0)	1 (0.6)
Missing	*625*	*189*	*436*
**Last HIV test (months)**			
< 6	458 (45.9)	230 (50.9)	255 (42.6)
[6 – 12[	239 (24.0)	98 (24.6)	141 (23.6)
≥ 12	182 (18.2)	69 (17.3)	113 (18.9)
Never	114 (11.4)	26 (6.5)	88 (14.7)
Does not know/does not want to answer	4 (0.4)	3 (0.7)	1 (0.2)
Missing	*3*	*1*	*2*
**Knowledge of boyfriend/husband's HIV status**			
Yes	121 (17.4)	33 (10.6)	88 (23.0)
No	573 (82.3)	279 (89.1)	294 (768)
Does not want to answer	2 (0.3)	1 (0.3)	1 (0.3)
Missing	*18*	*4*	*14*
**Interest in a medicine protecting against HIV**			
Yes	982 (98.6)	394 (99.0)	588 (98.3)
No	11 (1.1)	2 (0.5)	9 (1.5)
Does not know	3 (0.3)	2 (0.5)	1 (0.2)
Missing	*4*	*2*	*2*
**If yes, would agree to a medical follow-up every 3 months**			
Yes	964 (99.4)	391 (99.7)	573 (99.1)
No	5 (0.5)	1 (0.3)	4 (0.7)
Does not know	1 (0.1)	0 (0.0)	1 (0.2)
Missing	*12*	*2*	*10*

[1]This variable was added during the survey; for this reason, some participants did not answer the question.

Beyond HIV, many unmet SRH needs exist

In total, 43% of the survey participants reported at least one unwanted pregnancy, and 50% had at least one abortion in their lifetime (Table 3). Only 39% of surveyed FSWs were using a contraceptive method other than condoms; among them, most FSWs in Abidjan mentioned taking the pill (70%) compared to only 33% of FSWs in San Pedro, where 35% declared using an implant. Unfortunately, child desire was not asked in the quantitative survey, which does not allow us to calculate the unmet need for contraception among FSWs. However, as a proxy, it appeared that most of the interviewed FSWs did not want a child at the moment.

"My main risk, it is to not get pregnant because I'm still a schoolgirl. If I get pregnant, who will take care of it [the baby]*?"* (In-depth interview, Abidjan, 18 years old)

However, women explained during interviews that they feared becoming sterile because of contraceptive means, especially the pill.

"They [peer educators from the community-based NGO] told me about the pill, but I refused because I don't have children yet. I don't want to have problems in the future." (In-depth interview, Abidjan, 18 years old)

"That's what makes me tired. I'm afraid because I don't have children yet. That's my problem, otherwise for diseases, well, there are condoms." (In-depth interview, San Pedro, 19 years old)

Moreover, only half of the survey participants knew about emergency contraception, among which 36% knew only nonmedical means.

For peer review only - http://bmjopen.bmj.com/site/about/guidelines.xhtml

Additionally, 36% of survey participants practiced sex work during menstruation, mainly using tampons (62%) or cold water (24%) to stop the bleeding.

Finally, 79% of FSWs in San Pedro and 55% of FSWs in Abidjan reported contracting an STI over the past 12 months. Even though half of the questionnaire survey participants thought they were very exposed to HIV infection, the interviewed FSWs often declared being preoccupied by other diseases as well, such as STIs or cancer.

"But we, every time, when we go in the bush, it's not only AIDS that kills. There are several diseases. Today we talk about cervical cancer. So I think it's not only AIDS we should get protected from. We have to protect ourselves from many diseases that are sexual." (FGD, San Pedro)

Despite the work of peer educators at prostitution sites, few surveyed FSWs visited the dedicated community clinics, with 76% in San Pedro and 61% in Abidjan consulting a health practitioner over the past year, among which 40% in San Pedro and 17% in Abidjan went to a dedicated facility. In interviews, some FSWs reported the inconvenient opening times and/or location, the fear of being identified as a FSW in the clinic area and the stigmatizing and judgmental attitudes of health professionals as reasons for not visiting these clinics. In the event of condom breakage, FSWs usually relied on self-medication. For example, a young woman described the beverages she would use for a vaginal douche.

"I'm going to buy Coke with Nescafe. It's for cleaning everything falling down." (In-depth interview, Abidjan, 17 years old)

They act similarly in case of suspicion of an STI.

"*Before going to the clinic, we try traditional plants and medicines first*." (FGD, San Pedro)

Table 3. Sexual and reproductive health in the quantitative survey

Variables	All women n (%) n=1000	San Pedro n (%) n=400	Abidjan n (%) n=600
**Had at least one undesired pregnancy**			
Yes	416 (42.9)	173 (45.1)	243 (41.5)
No	554 (57.1)	211 (54.9)	343 (58.5)
Missing	*30*	*16*	*14*
**Had at least one abortion**			
Yes	488 (50.2)	195 (50.4)	293 (50.0)
No	485 (49.8)	192 (49.6)	293 (50.0)
Missing	*27*	*13*	*14*
**Use of contraception other than condom**			
Yes	391 (39.1)	193 (48.3)	198 (33.0)
No	608 (60.8)	206 (51.5)	402 (67.0)
Does not know	1 (0.1)	1 (0.2)	0 (0.0)
**If yes, which contraceptive method[2]**			
Pill	204 (52.0)	65 (33.5)	139 (70.2)
Injectable	91 (23.3)	55 (28.5)	36 (18.2)
Implant	83 (21.2)	68 (35.1)	15 (7.6)
Other method[1] (nivaquine, spice, traditional medicine, etc.)	24 (6.1)	13 (6.7)	11 (5.6)
**Knowledge of emergency contraception**			
Yes	472 (48.4)	195 (50.8)	277 (46.9)
No	497 (51.0)	183 (47.7)	314 (53.1)
Does not know	6 (0.6)	6 (1.5)	0 (0.0)
Missing	*25*	*16*	*9*
**If yes, type of emergency contraception known[2]**			
Morning-after pill	304 (64.1)	100 (49.7)	204 (74.7)
Other[1] (antibiotic, coffee, soda, salted water, and lemon)	170 (35.9)	101 (50.3)	69 (25.3)
**Sex work during menstruation**			
Yes	363 (36.4)	154 (38.5)	209 (34.9)
No	635 (63.6)	246 (61.5)	389 (65.1)
Missing	*2*	*0*	*2*

For peer review only - http://bmjopen.bmj.com/site/about/guidelines.xhtml

**If yes, tool used for sex work during menstruation[2]**			
Wash with ice-cold water	86 (24.0)	37 (24.2)	49 (23.7)
Piece of ice	26 (7.3)	11 (7.2)	15 (7.3)
Tampon	222 (62.0)	105 (68.6)	117 (57.1)
Other tools[1] (hot water, soapy water, cotton, etc.)	49 (13.7)	12 (7.8)	37 (17.9)
**Self-reported STI (last 12 months)**			
Yes	639 (64.7)	312 (78.8)	327 (55.2)
No	349 (35.3)	84 (21.2)	265 (44.8)
Missing	*12*	*4*	*8*
**Last medical consultation**			
Less than 3 months	195 (19.6)	101 (25.4)	94 (15.7)
3 – 12 months	475 (47.7)	202 (50.8)	273 (45.6)
More than a year	258 (25.9)	79 (19.8)	179 (29.9)
Never consulted	68 (6.8)	16 (4.0)	52 (8.7)
Missing	*4*	*2*	*2*
**If ever consulted, site of the last consultation with a doctor/nurse**			
Dedicated facility	225 (26.3)	141 (40.5)	84 (16.6)
Public facility	415 (48.6)	123 (35.3)	292 (57.7)
Private facility	213 (24.9)	83 (23.8)	130 (25.7)
Does not know	1 (0.1)	1 (0.3)	0 (0.0)
Missing	*74*	*34*	*40*

[1]"Other" categories describe participants' specific answers.
[2]Several possible answers.

DISCUSSION

Both the quantitative and qualitative results showed that FSWs were highly exposed to HIV despite their use of condoms. There were in fact a variety of situations in which the surveyed FSWs had condomless sex. First, the large majority did not use condoms with their regular partner despite their acknowledged concurrent sexual partnerships. Some women experienced coercion on the part of their male partners, questioning their faith in the relationship; having condomless sex was a proof of trust that was difficult to negotiate. Others used condomless sex as a negotiation strategy to obtain protection from their partners against the threat of violence. It seemed to be a calculated risk-mitigation strategy, although women were then exposed to the risk of HIV transmission. Second, some FSWs accepted condomless sexual intercourse for a large sum of money, especially when they had had few previous clients. Financial need associated with low prices of sexual intercourses and irregular weekly earnings drove some FSWs to engage in condomless sex as a way to earn more. In a context where gender norms reinforce male domination over women (20), they consciously took risks when facing the primacy of men's sexual pleasure (21). Third, the violence or the threat thereof that FSWs faced sometimes prevented them to negotiate condom use. Different studies showed that women who are victims of abuse are less likely to use condoms with their clients than those who are not (22,23). Performing an illegal activity can also compel women, especially in the street, to negotiate quickly with clients at the expense of condom use. The situation appeared even worse in some rural areas around San Pedro, where the interviewed FSWs revealed that they could not buy any condoms in the village, as there was no point of sale.

Despite the adoption of preventive behavior (condom use) in most cases, FSWs are still highly exposed to HIV, due to the occurrence of remaining unprotected sexual acts. In a complementary study within the PrEP-CI project (not yet published), we estimated the incidence among the 1000 surveyed FSWs using a recent infection testing algorithm adapted to the Ivorian context: we found an incidence of 2.2 per 100 person-years (1.5 in Abidjan and 3.2 in San Pedro) (24). In such context, oral PrEP could be an

For peer review only - http://bmjopen.bmj.com/site/about/guidelines.xhtml

appropriate and complementary preventive tool to cover the situations where condom use cannot be negotiated.

The majority of interviewed and surveyed FSWs had low awareness and knowledge of PrEP before our study but most of them were highly willing to use this medicine despite the constraint of regular medical follow-up. Participants felt PrEP would give added protection against infection, in particular with regular partners. A study in Kenya had similar findings and suggested to promote PrEP through outreach activities for sex workers (25). However, a PrEP implementation trial in South Africa showed low adherence despite high declared acceptability before the implementation (18). For this reason we were attentive to challenges that might hinder PrEP uptake and adherence for FSWs, such as side-effects or timing of daily administration.

Our results showed that FSWs faced many unmet needs regarding SRH beyond HIV prevention and treatment. Inconsistent condom use exposed FSWs to STIs (26,27) and undesired pregnancies (28), which could increase their mortality and morbidity (29). The prevalence of contraceptive use was low in the surveyed population despite their high risk of undesired pregnancy due to the common fear of contraception causing sterility (30). Furthermore, using ice or tissues to continue sex work during menstruation has been proven to be a source of bacterial infections (31). These needs could be addressed in the community clinics of the two NGOs. However, as shown in other studies, FSWs faced many obstacles in accessing SRH care, because of the high costs or distance of the sites (32), the stigmatizing and discriminating attitudes of some health practitioners, the FSWs' social and economic marginalization, and restrictive laws related to their activity (33). FSWs thus preferred advice from their peers or self-medication. Moreover, peer educators from both NGOs expressed that public policies and international donors in Côte d'Ivoire currently focus on the identification and referral of new cases of HIV-positive FSWs, while HIV-negative women have limited access to care as stated above. A PrEP program requires a medical follow-up every three months and thus implies to consider the chronic follow-up of HIV-negative women. All efficacy PrEP trials provided a range of sexual healthcare services in addition to PrEP drugs. By design, these services were conditional to PrEP use. When transitioning to real life, such PrEP programs reproduced such service model. Our results suggest that a paradigm shift toward a patient-centered approach should be preferred, that is offering sexual and reproductive health services (such as contraception or STI testing and treatment) in which PrEP is an option but not mandatory. SRH services could also be a way to engage FSWs not ready for PrEP into regular care.

In addition, FSWs in San Pedro appeared to be in a more precarious situation than those in Abidjan due to their lower education level, higher number of children, irregularity of work, multiplicity of clients and work locations, and the lower price of sexual intercourse. They were also more likely to have condomless intercourse, notably for a large sum of money, and to report having had an STI over the past year. This can be explained by the fact that a large percentage of FSWs in San Pedro came to the area during the period of coffee and cocoa exploitation (September-December), which brought many migrant workers; this results in less stability and security. The high mobility of these women generates "seasons of risk" (34), i.e., times when an individual might face an increased risk of HIV infection. It is paramount to take this into account when implementing daily PrEP for these women (35); they are the population most likely to regularly suspend their PrEP use. Developing mobile clinics that deliver HIV and SRH care services directly at prostitution sites could mitigate the issue of FSWs' mobility and address barriers in access to care, such as distance between prostitution sites and clinics or stigmatization associated with their activity.

FSWs' needs for PrEP cannot be understood without additionally considering the broader contexts in which their risk of exposure to HIV is situated: the context of their work, their relationships and their

For peer review only - http://bmjopen.bmj.com/site/about/guidelines.xhtml

concerns about family planning or stigmatization, etc. This is important information to consider if a PrEP program is to successfully serve this at-risk population. In order to address women's experiences and concerns, a global SRH care package delivered through both community clinics and mobile clinics on prostitution sites appear essential. Several studies related to PrEP and SRH needs of FSWs showed as well that combination prevention approaches are necessary. First, as pointed by a study in Zimbabwe (36), women need to perceive the risk of getting infected by HIV and to be able to access health services in order to take PrEP daily. Second, as shown by Dhana *et al.* in a systematic review (37), there is a lack of coordination between SRH and HIV services dedicated to FSWs in Africa when they are two distinct services; HIV services delivery models should integrate SRH services. Furthermore, in order to minimize stigma related to entry into care, services for HIV-positive and services for HIV-negative should not be dissociated.

A strength of this study is the use of mixed-method allowing us to better describe and understand challenges of PrEP implementation among FSWs in Côte d'Ivoire, as well as the strong collaboration with two NGOs helping us to reach the FSWs. Yet, this study has some limitations. First, as it focused on FSWs reached by two NGOs, the included population was probably more likely to know about HIV prevention and to access SRH care. In addition, our sample did not include occasional or undeclared FSWs. As such, the results cannot be extrapolated to all FSWs working inside or around Abidjan and San Pedro but can provide an operational perspective for developing healthcare services. Peer-educators conducted the survey during their on-site activities, which already consistently extended their working time. For logistic reasons, it was not possible to monitor the number of FSWs present on site, potentially eligible, examined for eligibility and included in the survey. Therefore we are not able to provide participation rates. However, peer educators reported that most FSWs confirmed for eligibility did accept to answer the questionnaire. The survey was stopped when we reached the expected number of 1000 FSWs (600 in Abidjan and 400 in San Pedro) and all FSWs included in the survey were analyzed. Finally, the use of FGDs could have led to social desirable answers. A matter of concern, pointed out by field workers and data collected, are the young underage FSWs (three interviews were conducted with FSWs aged less than 18 on the beaches of Abidjan). Ivorian law authorizes HIV testing for teenagers aged 16 and 17, without parental consent. What about other care and services that cannot be delivered to them in the absence of consent?

CONCLUSIONS

Implementing PrEP among FSWs in West Africa, such as in Côte d'Ivoire, is not only about providing a new prevention tool but is also an invitation to consider the chronic follow-up of HIV-negative FSWs. A global care package should be offered to FSWs, including HIV prevention and care, STI screening and treatment, contraception, menstrual management counseling and HBV screening, vaccination and medical treatment. In addition, PrEP initiation should not limit access to SRH services; conversely, SRH services could be a way to attract FSWs to HIV prevention. Beyond reducing the risk of HIV among FSWs and their partners, PrEP provides an opportunity to improve their health condition more globally.

While current policies focus on only HIV-infected women and on the importance of testing new FSWs, our results highlight the importance of developing a people-focused approach, as opposed to an "HIV-focused approach", that integrates all SRH needs when transitioning from PrEP efficacy trials to implementation (19).

6. Conflict of interest statement

There are no conflicts of interest.

For peer review only - http://bmjopen.bmj.com/site/about/guidelines.xhtml

7. Contributorship Statement

JL, SE and CD designed the ANRS 12361 PrEP-CI study. JMM and MN implemented the quantitative survey with the support of CA and SK. VB conducted the qualitative interviews. VB and JL developed the research question addressed in this paper. VB did the qualitative analysis, and MN did the statistical analysis. VB wrote the manuscript with the support of JL, MP and MN. All authors contributed to the interpretation and presentation of the findings. All authors approved the final version of the manuscript for submission.

8. Funding

The PrEP-CI ANRS 12361 was funded by the Bill and Melinda Gates Foundation (Investment ID: OPP1106343) and the French National Agency for AIDS and Viral Hepatitis Research (ANRS).

9. Acknowledgments

We would like to thank all participants as well as Aprosam's and Espace Confiance's peer educators.

10. Data sharing statement

Dataset are available upon request on Zenodo (DOI: 10.5281/zenodo.2269160).

11. References

1. Dwyer-Lindgren L, Cork MA, Sligar A, Steuben KM, Wilson KF, Provost NR, et al. Mapping HIV prevalence in sub-Saharan Africa between 2000 and 2017. Nature. 2019 Jun;570(7760):189–93.
2. Kharsany ABM, Karim QA. HIV Infection and AIDS in Sub-Saharan Africa: Current Status, Challenges and Opportunities. Open AIDS J. 2016 Apr 8;10:34–48.
3. Baggaley R, Hensen B, Ajose O, Grabbe K, Wong V, Schilsky A, et al. From caution to urgency: the evolution of HIV testing and counselling in Africa. Bulletin of the World Health Organization. 2012 Sep 1;90(9):652-658B.
4. World Health Organization. Consolidated Guidelines on Hiv Prevention, Diagnosis, Treatment and Care for Key Populations [Internet]. World Health Organization; 2016 [cited 2019 Sep 26]. Available from: http://proxy.library.carleton.ca/loginurl=https://www.deslibris.ca/ID/10063272
5. World Health Organization. Consolidated Guidelines on the Use of Antiretroviral Drugs for treating and preventing HIV infection. Recommendations for a Public Health Approach. Second Edition 2016. World Health Organization; 2016 p. 429.
6. UNAIDS. Factsheet - Global AIDS Update 2019. 2019 p. 6.
7. Molina J-M, Capitant C, Spire B, Pialoux G, Cotte L, Charreau I, et al. On-Demand Preexposure Prophylaxis in Men at High Risk for HIV-1 Infection. New England Journal of Medicine. 2015 Dec 3;373(23):2237–46.
8. Grant RM, Lama JR, Anderson PL, McMahan V, Liu AY, Vargas L, et al. Preexposure chemoprophylaxis for HIV prevention in men who have sex with men. N Engl J Med. 2010 Dec 30;363(27):2587–99.
9. Baeten JM, Donnell D, Ndase P, Mugo NR, Campbell JD, Wangisi J, et al. Antiretroviral Prophylaxis for HIV Prevention in Heterosexual Men and Women. New England Journal of Medicine. 2012 Aug 2;367(5):399–410.
10. WHO. Guideline on when to start antiretroviral therapy and on pre-exposure prophylaxis for HIV [Internet]. Geneva: World Health Organisation; 2015 Sep p. 78. Report No.: 978 92 4 150956 5. Available from: http://apps.who.int/iris/bitstream/10665/186275/1/9789241509565_eng.pdf
11. UNAIDS. UNAIDS data 2017 [Internet]. UNAIDS; 2017 [cited 2018 Jul 11]. Available from: http://www.unaids.org/sites/default/files/media_asset/2017_data-book_en.pdf

For peer review only - http://bmjopen.bmj.com/site/about/guidelines.xhtml

12. UNAIDS. On the Fast-Track to end AIDS by 2030: Focus on location and population [Internet]. 2015 p. 268. Available from: http://www.unaids.org/en/resources/documents/2015/FocusLocationPopulation
13. Corneli AL, Deese J, Wang M, Taylor D, Ahmed K, Agot K, et al. FEM-PrEP: Adherence Patterns and Factors Associated With Adherence to a Daily Oral Study Product for Pre-exposure Prophylaxis. J Acquir Immune Defic Syndr. 2014 Jul 1;66(3):324–31.
14. Marrazzo JM, Ramjee G, Richardson BA, Gomez K, Mgodi N, Nair G, et al. Tenofovir-based preexposure prophylaxis for HIV infection among African women. N Engl J Med. 2015 Feb 5;372(6):509–18.
15. Béhanzin L, Guédou FA, Geraldo N, Goma Matsétsé E, Aza-Gnandji M, Imorou Bah Chabi A, et al. PrEP and Early Antiretroviral Therapy Demonstration Project: Challenges to Ensure Follow-up and Adherence Among Female Sex Workers in Cotonou, Benin. In Chicago: HIVR4P 2016 Conference; 2016.
16. Alary M, Béhanzin L, Mboup A, Guédou FA, Geraldo N, Goma-Matsétsé E, et al. Early Antiretroviral Therapy and Daily Pre-exposure Prophylaxis for HIV Prevention Among Female Sex Workers in Cotonou, Benin: A Demonstration Study. In Madrid: HIVR4P 2016 Conference; 2018.
17. Eakle R, Gomez GB, Naicker N, Bothma R, Mbogua J, Cabrera Escobar MA, et al. HIV pre-exposure prophylaxis and early antiretroviral treatment among female sex workers in South Africa: Results from a prospective observational demonstration project. PLoS Med. 2017;14(11):1–17.
18. Eakle R, Bourne A, Mbogua J, Mutanha N, Rees H. Exploring acceptability of oral PrEP prior to implementation among female sex workers in South Africa. J Intern AIDS Soc. 2018 Feb 1;21(2):n/a-n/a.
19. Larmarange J, Becquet V, Masumbuko J-M, Nouaman M, Plazy M, Danel C, et al. Implementing preexposure prophylaxis among key populations: an opportunity for patient-centered services and management of hepatitis B. AIDS. 2018 Mar 27;32(6):829–30.
20. Deering KN, Shaw SY, Thompson LH, Ramanaik S, Raghavendra T, Doddamane M, et al. Fertility intentions, power relations and condom use within intimate and other non-paying partnerships of women in sex work in Bagalkot District, South India. AIDS Care. 2015 Oct 3;27(10):1241–9.
21. Ghimire L, Smith WCS, van Teijlingen ER, Dahal R, Luitel NP. Reasons for non- use of condoms and self- efficacy among female sex workers: a qualitative study in Nepal. BMC Women's Health [Internet]. 2011 Sep 26;11(42). Available from: https://doi.org/10.1186/1472-6874-11-42
22. Wirtz AL, Schwartz S, Ketende S, Anato S, Nadedjo FD, Ouedraogo HG, et al. Sexual Violence, Condom Negotiation, and Condom Use in the Context of Sex Work: Results From Two West African Countries. JAIDS Journal of Acquired Immune Deficiency Syndromes. 2015 Mar;68:S171.
23. Decker MR, McCauley HL, Phuengsamran D, Janyam S, Seage GR, Silverman JG. Violence victimisation, sexual risk and sexually transmitted infection symptoms among female sex workers in Thailand. Sexually Transmitted Infections. 2010 Jun 1;86(3):236–40.
24. Nouaman MN, Becquet V, Masumbuko J-M, Anoma C, Soh K, Plazy M, et al. Évaluation de l'incidence du VIH chez des travailleuses du sexe en Côte d'Ivoire (PrEP-CI ANRS 12361). In Bordeaux; 2018.
25. Restar AJ, Tocco JU, Mantell JE, Lafort Y, Gichangi P, Masvawure TB, et al. Perspectives on HIV Pre- and Post- Exposure Prophylaxes (PrEP and PEP) among Female and Male Sex Workers in Mombasa, Kenya: Implications for Integrating Biomedical Prevention into Sexual Health Services. AIDS Educ Prev. 2017 Apr;29(2):141–53.
26. Braunstein SL, Ingabire CM, Kestelyn E, Uwizera AU, Mwamarangwe L, Ntirushwa J, et al. High Human Immunodeficiency Virus Incidence in a Cohort of Rwandan Female Sex Workers. Sexually Transmitted Diseases. 2011 May;38(5):385.
27. Scorgie F, Chersich MF, Ntaganira I, Gerbase A, Lule F, Lo Y-R. Socio-Demographic Characteristics and Behavioral Risk Factors of Female Sex Workers in Sub-Saharan Africa: A Systematic Review. AIDS Behav. 2012 May 1;16(4):920–33.
28. Schwartz S, Papworth E, Thiam-Niangoin M, Abo K, Drame F, Diouf D, et al. An urgent need for integration of family planning services into HIV care: the high burden of unplanned pregnancy,

For peer review only

For peer review only - http://bmjopen.bmj.com/site/about/guidelines.xhtml

termination of pregnancy, and limited contraception use among female sex workers in Côte d'Ivoire. JAIDS Journal of Acquired Immune Deficiency Syndromes. 2015 Mar 1;68(Supplement 3):S91–S98.

29. Mullick S. Sexually transmitted infections in pregnancy: prevalence, impact on pregnancy outcomes, and approach to treatment in developing countries. Sexually Transmitted Infections. 2005 Aug 1;81(4):294–302.
30. Guillaume A, Ltd. A. The Role of Abortion in the Fertility Transition in Abidjan (Côte d'Ivoire) during the 1990s. Population (English Edition, 2002-). 2003;58(6):657–85.
31. Baisley K, Changalucha J, Weiss HA, Mugeye K, Everett D, Hambleton I, et al. Bacterial vaginosis in female facility workers in north-western Tanzania: prevalence and risk factors. Sexually Transmitted Infections. 2009 Sep 1;85(5):370–5.
32. Wahed T, Alam A, Sultana S, Rahman M, Alam N, Martens M, et al. Barriers to sexual and reproductive healthcare services as experienced by female sex workers and service providers in Dhaka city, Bangladesh. PLoS ONE. 2017 31;12(7).
33. Ippoliti NB, Nanda G, Wilcher R. Meeting the Reproductive Health Needs of Female Key Populations Affected by HIV in Low-and Middle-Income Countries: A Review of the Evidence. Studies in Family Planning. 2017;48(2):121–151.
34. Elsesser SA, Oldenburg CE, Biello KB, Mimiaga MJ, Safren SA, Egan JE, et al. Seasons of Risk: Anticipated Behavior on Vacation and Interest in Episodic Antiretroviral Pre-exposure Prophylaxis (PrEP) Among a Large National Sample of U.S. Men Who have Sex with Men (MSM). AIDS Behav. 2016 Jul;20(7):1400–7.
35. Namey E, Agot K, Ahmed K, Odhiambo J, Skhosana J, Guest G, et al. When and why women might suspend PrEP use according to perceived seasons of risk: implications for PrEP-specific risk-reduction counselling. Culture, Health & Sexuality. 2016 Sep;18(9):1081–91.
36. Cowan FM, Delany-Moretlwe S. Promise and pitfalls of pre-exposure prophylaxis for female sex workers. Curr Opin HIV AIDS. 2016 Jan;11(1):27–34.
37. Dhana A, Luchters S, Moore L, Lafort Y, Roy A, Scorgie F, et al. Systematic review of facility-based sexual and reproductive health services for female sex workers in Africa. Globalization and Health. 2014 Jun 10;10:46.

For peer review only - http://bmjopen.bmj.com/site/about/guidelines.xhtml

STROBE Statement—Checklist of items that should be included in reports of ***cross-sectional studies***

	Item No	Recommendation	Reported on page #
**Title and abstract**	1	(*a*) Indicate the study's design with a commonly used term in the title or the abstract	1
		(*b*) Provide in the abstract an informative and balanced summary of what was done and what was found	3
**Introduction**			
Background/rationale	2	Explain the scientific background and rationale for the investigation being reported	4
Objectives	3	State specific objectives, including any prespecified hypotheses	4
**Methods**			
Study design	4	Present key elements of study design early in the paper	5
Setting	5	Describe the setting, locations, and relevant dates, including periods of recruitment, exposure, follow-up, and data collection	5
Participants	6	(*a*) Give the eligibility criteria, and the sources and methods of selection of participants	5-6
Variables	7	Clearly define all outcomes, exposures, predictors, potential confounders, and effect modifiers. Give diagnostic criteria, if applicable	5
Data sources/ measurement	8*	For each variable of interest, give sources of data and details of methods of assessment (measurement). Describe comparability of assessment methods if there is more than one group	5-6
Bias	9	Describe any efforts to address potential sources of bias	
Study size	10	Explain how the study size was arrived at	14
Quantitative variables	11	Explain how quantitative variables were handled in the analyses. If applicable, describe which groupings were chosen and why	5
Statistical methods	12	(*a*) Describe all statistical methods, including those used to control for confounding	5
		(*b*) Describe any methods used to examine subgroups and interactions	5
		(*c*) Explain how missing data were addressed	5
		(*d*) If applicable, describe analytical methods taking account of sampling strategy	
		(*e*) Describe any sensitivity analyses	
**Results**			
Participants	13*	(a) Report numbers of individuals at each stage of study—eg numbers potentially eligible, examined for eligibility, confirmed eligible, included in the study, completing follow-up, and analysed	14
		(b) Give reasons for non-participation at each stage	14
		(c) Consider use of a flow diagram	
Descriptive data	14*	(a) Give characteristics of study participants (eg demographic, clinical, social) and information on exposures and potential confounders	7-8
		(b) Indicate number of participants with missing data for each variable of interest	Cf. tables
Outcome data	15*	Report numbers of outcome events or summary measures	
Main results	16	(*a*) Give unadjusted estimates and, if applicable, confounder-adjusted estimates and their precision (eg, 95% confidence interval). Make clear which confounders were adjusted for and why they were included	Cf. tables

For peer review only - http://bmjopen.bmj.com/site/about/guidelines.xhtml

		(*b*) Report category boundaries when continuous variables were categorized	Cf. tables
		(*c*) If relevant, consider translating estimates of relative risk into absolute risk for a meaningful time period	
Other analyses	17	Report other analyses done—eg analyses of subgroups and interactions, and sensitivity analyses	
**Discussion**			
Key results	18	Summarise key results with reference to study objectives	12-13
Limitations	19	Discuss limitations of the study, taking into account sources of potential bias or imprecision. Discuss both direction and magnitude of any potential bias	14
Interpretation	20	Give a cautious overall interpretation of results considering objectives, limitations, multiplicity of analyses, results from similar studies, and other relevant evidence	13-14
Generalisability	21	Discuss the generalisability (external validity) of the study results	14
**Other information**			
Funding	22	Give the source of funding and the role of the funders for the present study and, if applicable, for the original study on which the present article is based	15

*Give information separately for exposed and unexposed groups.

Note: An Explanation and Elaboration article discusses each checklist item and gives methodological background and published examples of transparent reporting. The STROBE checklist is best used in conjunction with this article (freely available on the Web sites of PLoS Medicine at http://www.plosmedicine.org/, Annals of Internal Medicine at http://www.annals.org/, and Epidemiology at http://www.epidem.com/). Information on the STROBE Initiative is available at www.strobe-statement.org.

For peer review only

For peer review only - http://bmjopen.bmj.com/site/about/guidelines.xhtml
